# Supplementary material for: A feedback loop between the androgen receptor and 6-phosphogluoconate dehydrogenase (6PGD) drives prostate cancer growth
Source: eLife. 2021 Aug 12;10:e62592. doi: 10.7554/eLife.62592 (PMC8416027; doi:10.7554/eLife.62592)
Supplement: Supplementary file 1. [file elife-62592-supp1.docx]

**Supplementary File 1. Primers used for quantitative Reverse Transcription PCR (qRT-PCR).**

| Target name | Forward (top) and reverse (bottom) primers |
| --- | --- |
| *AR* | CAACTCCTTCAGCAACAGCA |
|  | TCGAAGTGCCCCCTAAGTAA |
| *FKBP5* | AAAAGGCCAAGGAGCACAAC |
|  | TTGAGGAGGGGCCGAGTTC |
| *GAPDH* | TGCACCACCAACTGCTTAGC |
|  | GGCATGGACTGTGGTCATGAG |
| *GUSB* | CGTCCCACCTAGAATCTGCT |
|  | TTGCTCACAAAGGTCACAGG |
| *KLK2* | GGTGGCTGTGTACAGTCATGGAT |
|  | TGTCTTCAGGCTCAAACAGGTTG |
| *KLK3* | ACCAGAGGAGTTCTTGACCCCAAA |
|  | CCCCAGAATCACCCGAGCAG |
| *L19* | TGCCAGTGGAAAAATCAGCCA |
|  | CAAAGCAAATCTCGACACCTTG |
| *PGD* | CACAGCAGGGTTCTCCAGTT |
|  | GTCAGTGGTGGAGAGGAAGG |
| *PPIA* | GCATACGGGTCCTGGCAT |
|  | ACATGCTTGCCATCCAACC |
| *TMPRSS2* | GACCAAGAACAATGACATTGCG |
|  | GTTCTGGCTGCAGCATCATG |
| *TUBA1B* | CCTTCGCCTCCTAATCCCTA |
|  | CCGTGTTCCAGGCAGTAGA |
