# Supplementary material for: A feedback loop between the androgen receptor and 6-phosphogluoconate dehydrogenase (6PGD) drives prostate cancer growth
Source: eLife. 2021 Aug 12;10:e62592. doi: 10.7554/eLife.62592 (PMC8416027; doi:10.7554/eLife.62592)
Supplement: Supplementary file 2. [file elife-62592-supp2.docx]

**Supplementary File 2. Antibodies used for Western blotting and immunohistochemistry (IHC).**

| **Antibody** | **Dilution** | | **Supplier** | **Catalogue number** |
| --- | --- | --- | --- | --- |
|  | **Western blotting** | **IHC** |  |  |
| ACC-1 [C83B10] | 1:1000 |  | Cell Signaling Technology, Inc | 3676 |
| pACC-1 [Ser79] | 1:1000 |  | Cell Signaling Technology, Inc | 3661 |
| β-Actin (AC-15) | 1:1000 |  | Sigma Aldrich | A5441 |
| AR-N20 | 1:1000 |  | Santa Cruz Biotechnology Inc | SC-816 |
| AR |  | 1:200 | Abcam | ab108341 |
| AMPKα | 1:1000 |  | Cell Signaling Technology, Inc | 2532 |
| pAMPKα [Thr172]  40H9] | 1:1000 |  | Cell Signaling Technology, Inc | 2535 |
| GAPDH | 1:1000 |  | BioRad | 12004168 |
| Hsp90 | 1:1000 |  | Cell Signaling Technology, Inc | 4874 |
| Ki67 |  | 1:200 | Agilent Technologies | M724001-2 |
| P70 S6 Kinase (49D7) | 1:1000 |  | Cell Signaling Technology, Inc | 2708 |
| pP70 S6 Kinase  [Thr389] | 1:2000 |  | Cell Signaling Technology, Inc | 9205S |
| 6PGD | 1:1000 |  | ThermoFisher Scientific | PA5-21376 |
| 6PGD |  | 1:800 | Sigma Aldrich | HPA031314 |
| PSA | 1:1000 |  | ProteinTech Group | 10679-1-AP |
| S6 (5G10) | 1:1000 |  | Cell Signaling Technology, Inc | 2217 |
| pS6 [Ser235/236] | 1:1000 | 1:200 | Cell Signaling Technology, Inc | 2211 |
| Ubiquitin | 1:1000 |  | Genesearch | 3936 |
| Goat Anti-Rabbit  (Biotinylated) |  | 1:400 | Agilent Technologies | E043201-8 |
