# Supplementary material for: A feedback loop between the androgen receptor and 6-phosphogluoconate dehydrogenase (6PGD) drives prostate cancer growth
Source: eLife. 2021 Aug 12;10:e62592. doi: 10.7554/eLife.62592 (PMC8416027; doi:10.7554/eLife.62592)
Supplement: Source data 1. [file elife-62592-supp3.zip › Figures-Western blot Source data combined.pptx]

## Slide 1
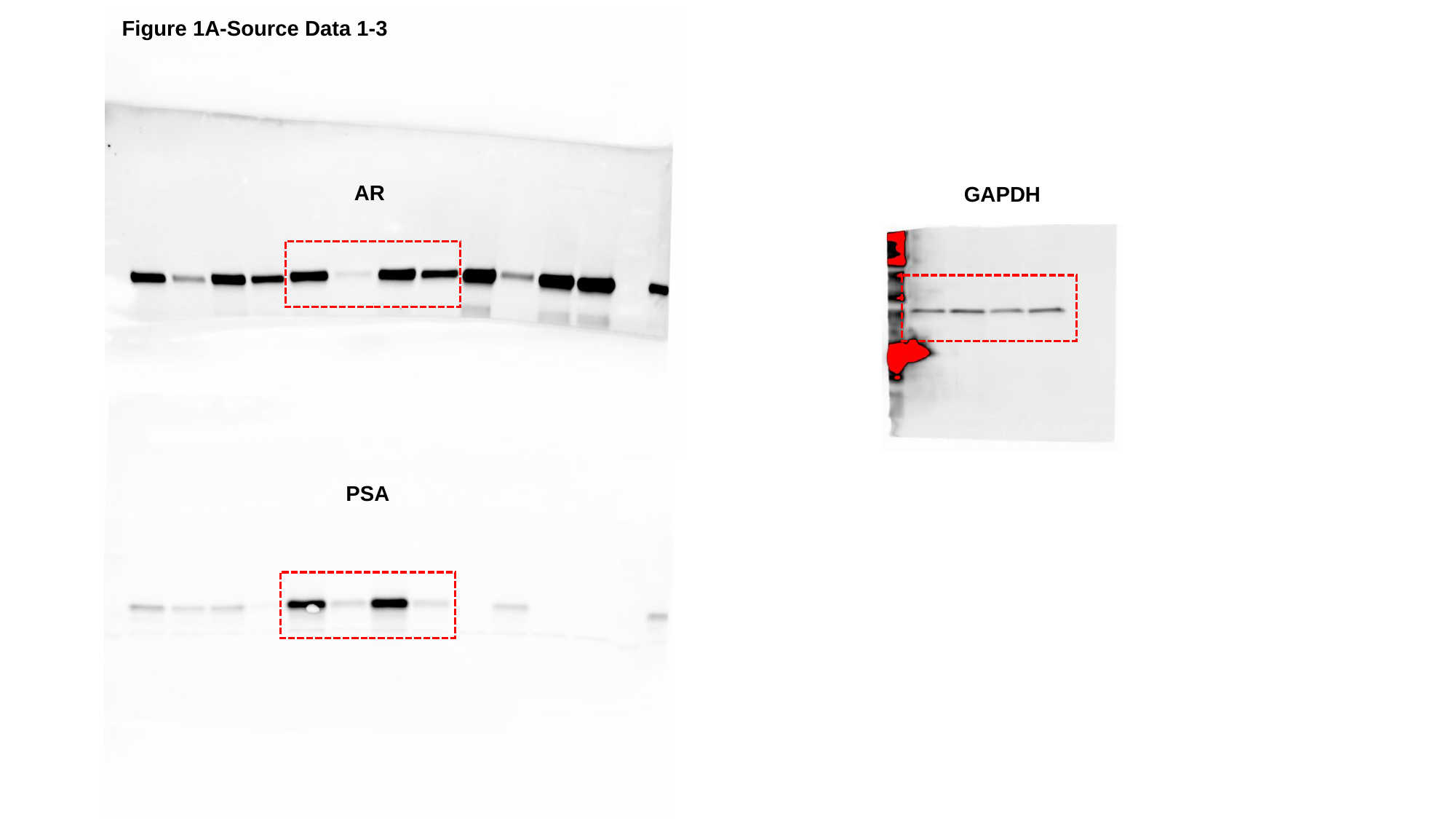

Figure 1A-Source Data 1-3
AR
GAPDH
PSA

## Slide 2
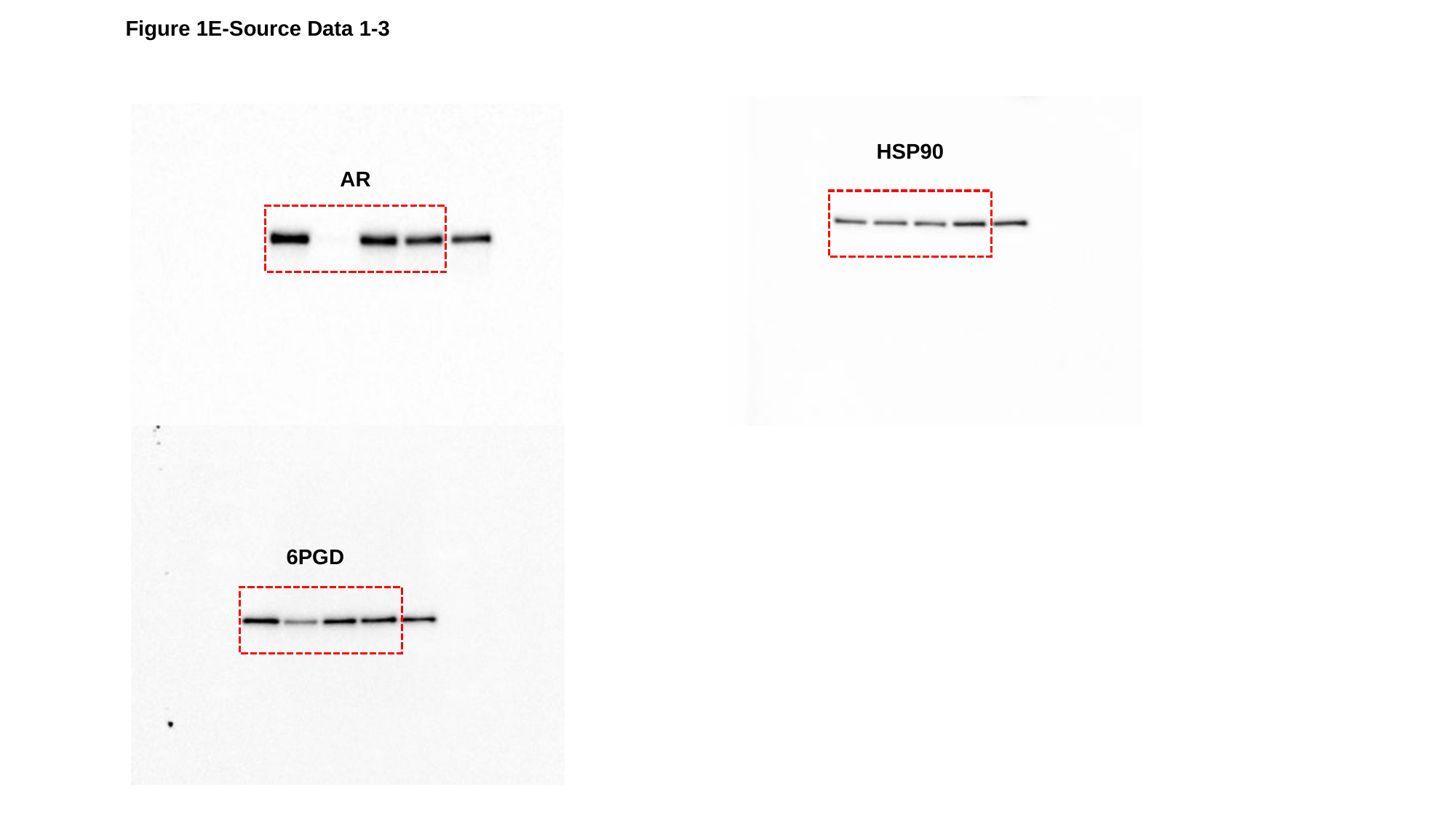

Figure 1E-Source Data 1-3
HSP90
AR
6PGD

## Slide 3
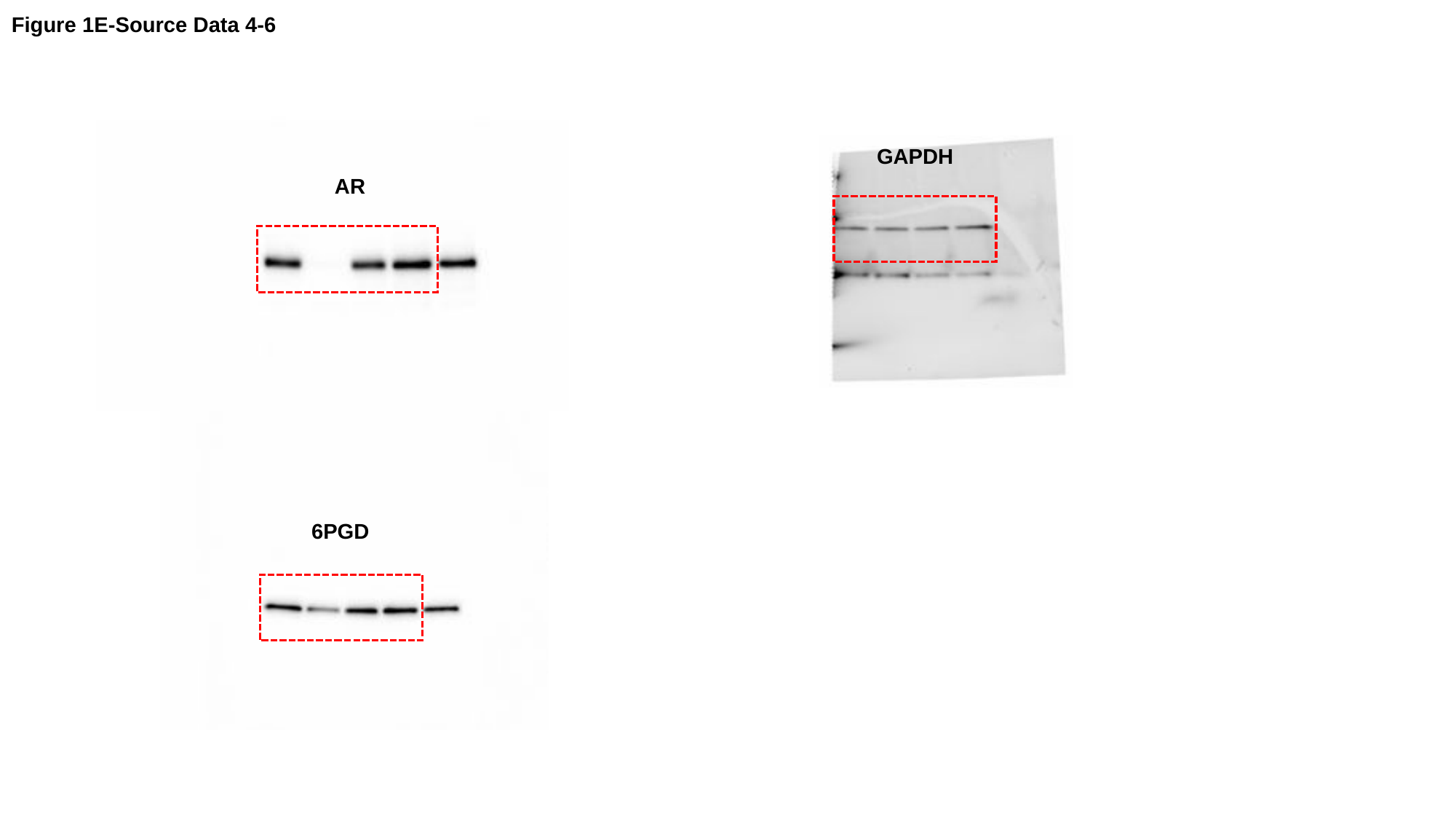

Figure 1E-Source Data 4-6
GAPDH
AR
6PGD

## Slide 4
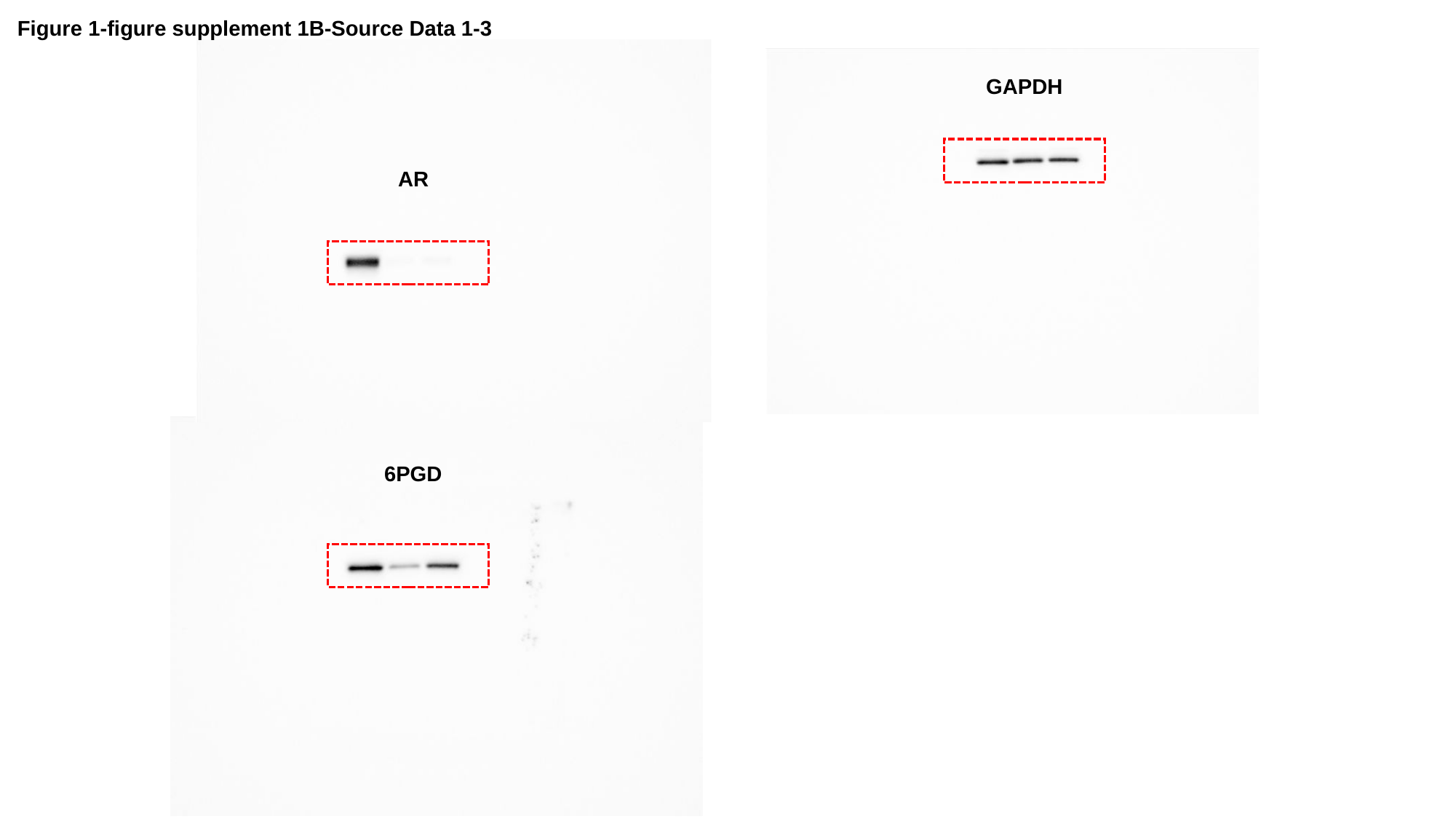

Figure 1-figure supplement 1B-Source Data 1-3
GAPDH
AR
6PGD

## Slide 5
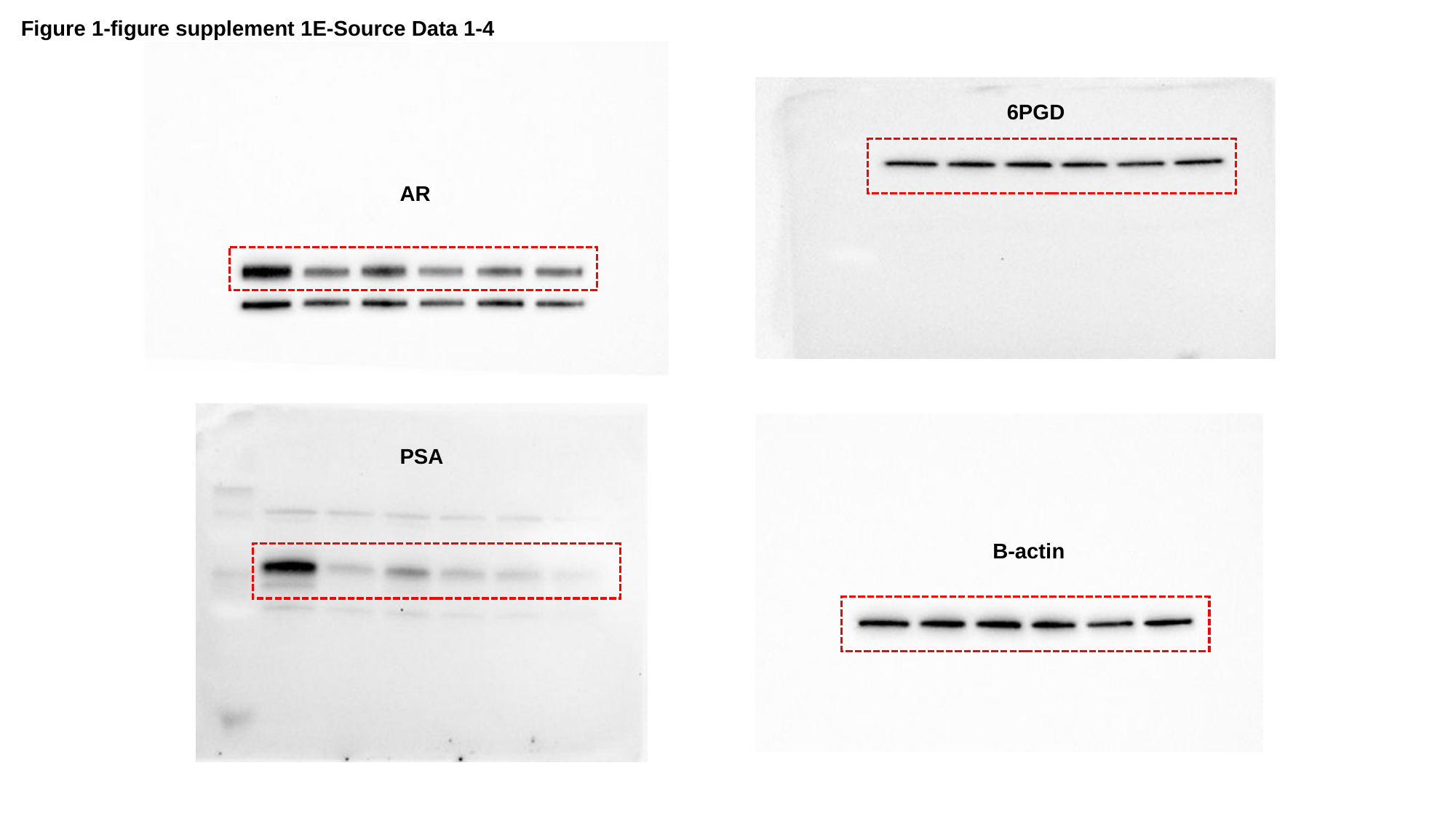

Figure 1-figure supplement 1E-Source Data 1-4
6PGD
AR
PSA
B-actin

## Slide 6
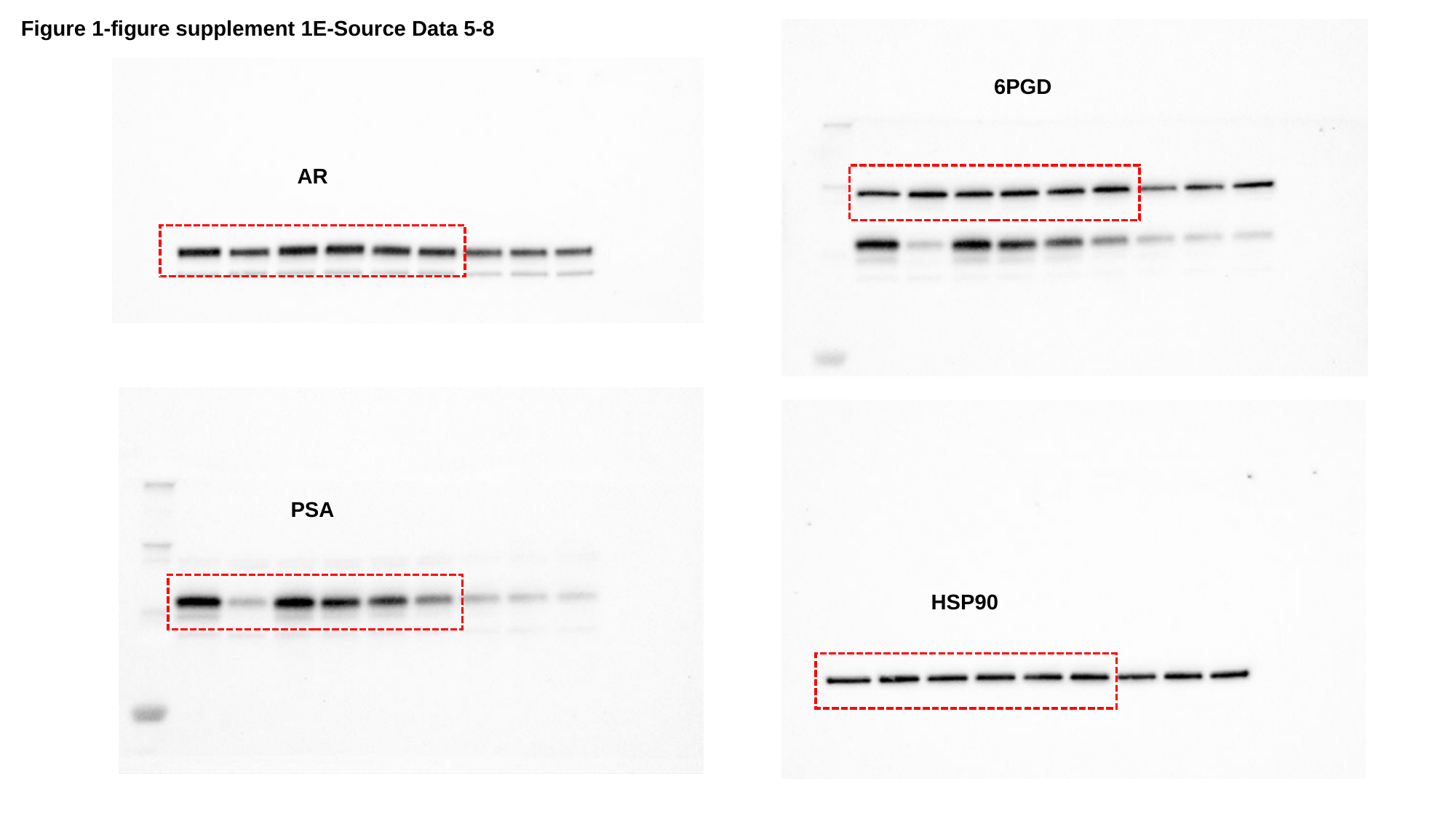

Figure 1-figure supplement 1E-Source Data 5-8
6PGD
AR
PSA
HSP90

## Slide 7
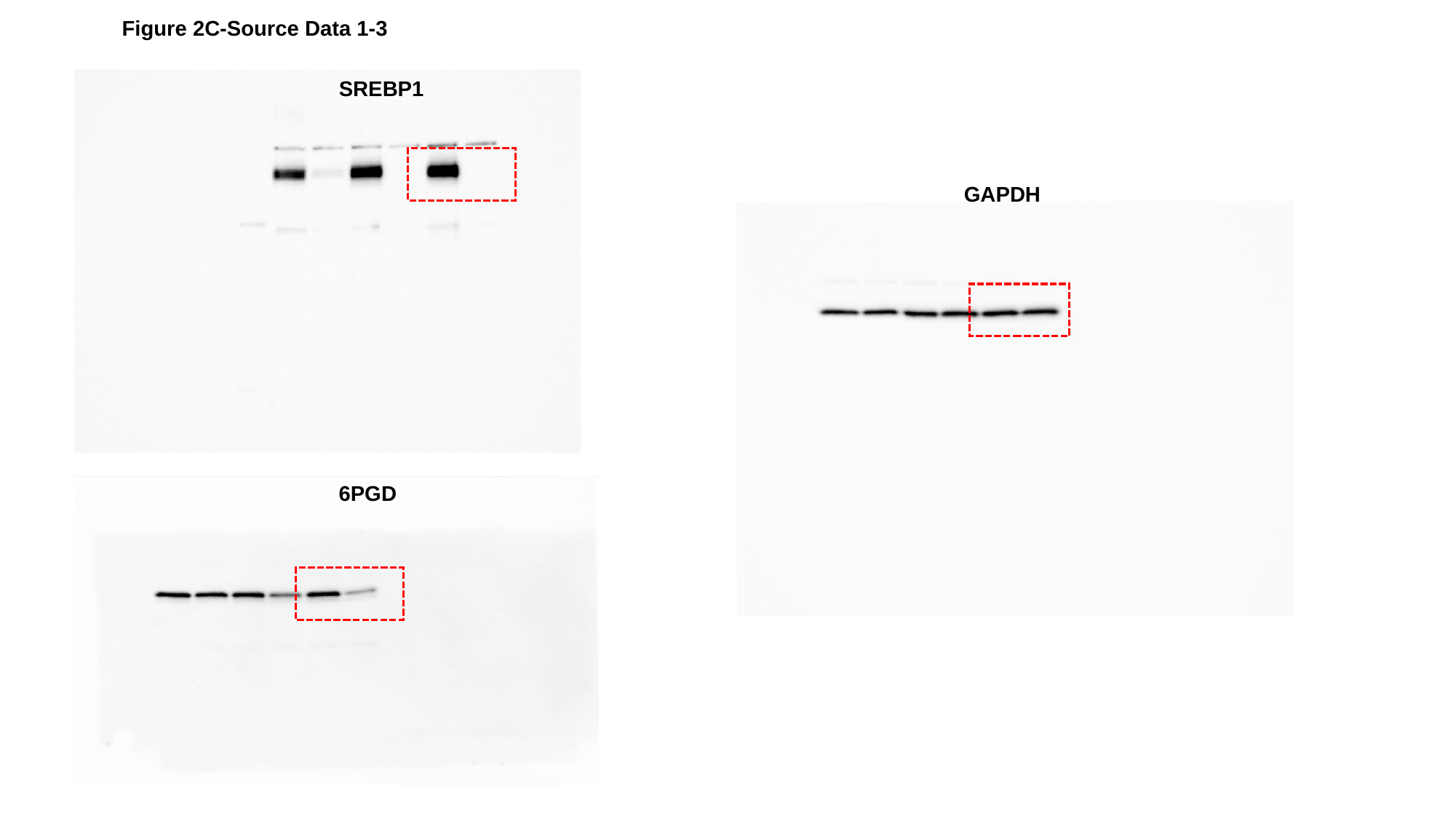

Figure 2C-Source Data 1-3
SREBP1
GAPDH
6PGD

## Slide 8
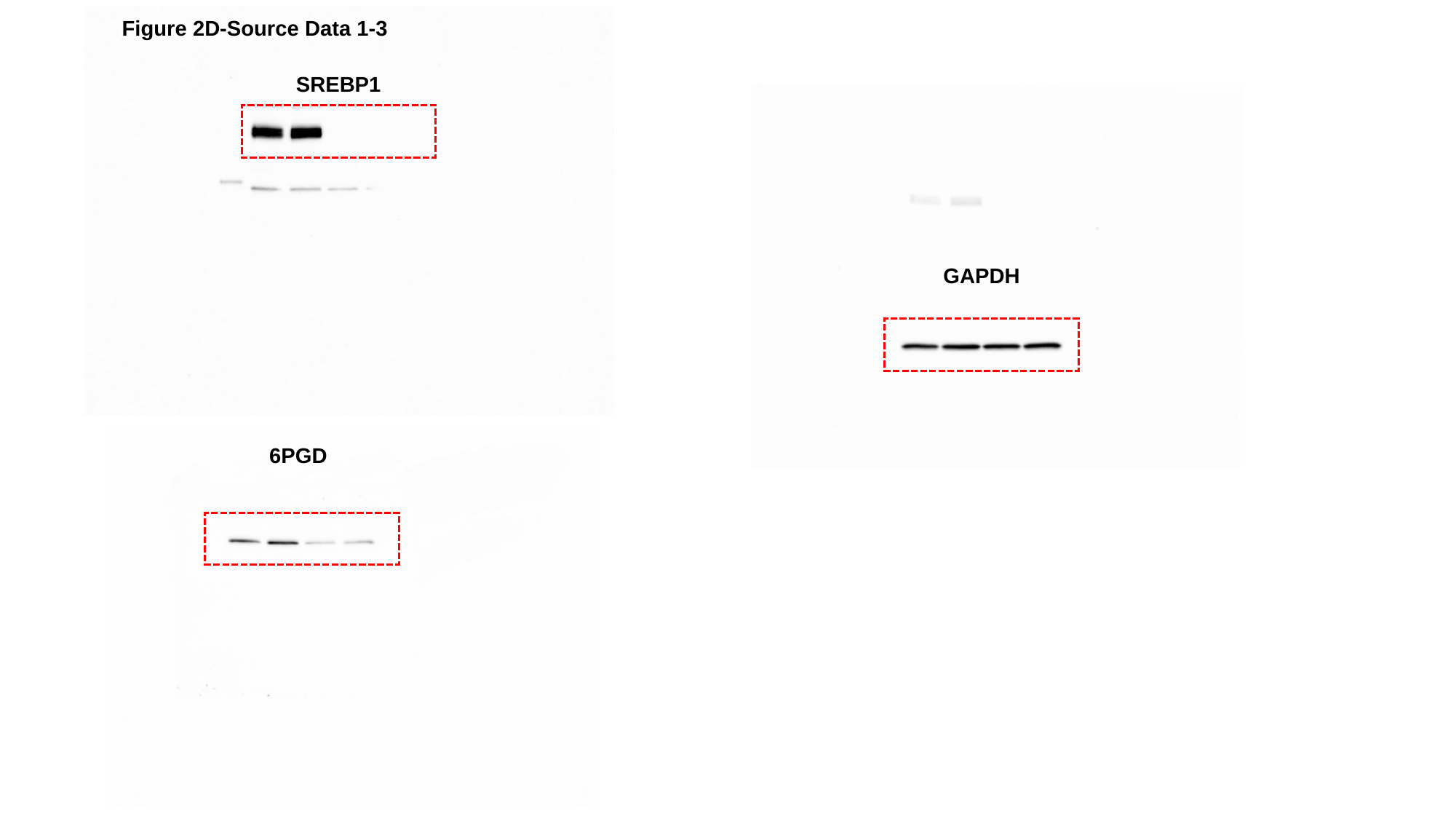

Figure 2D-Source Data 1-3
SREBP1
GAPDH
6PGD

## Slide 9
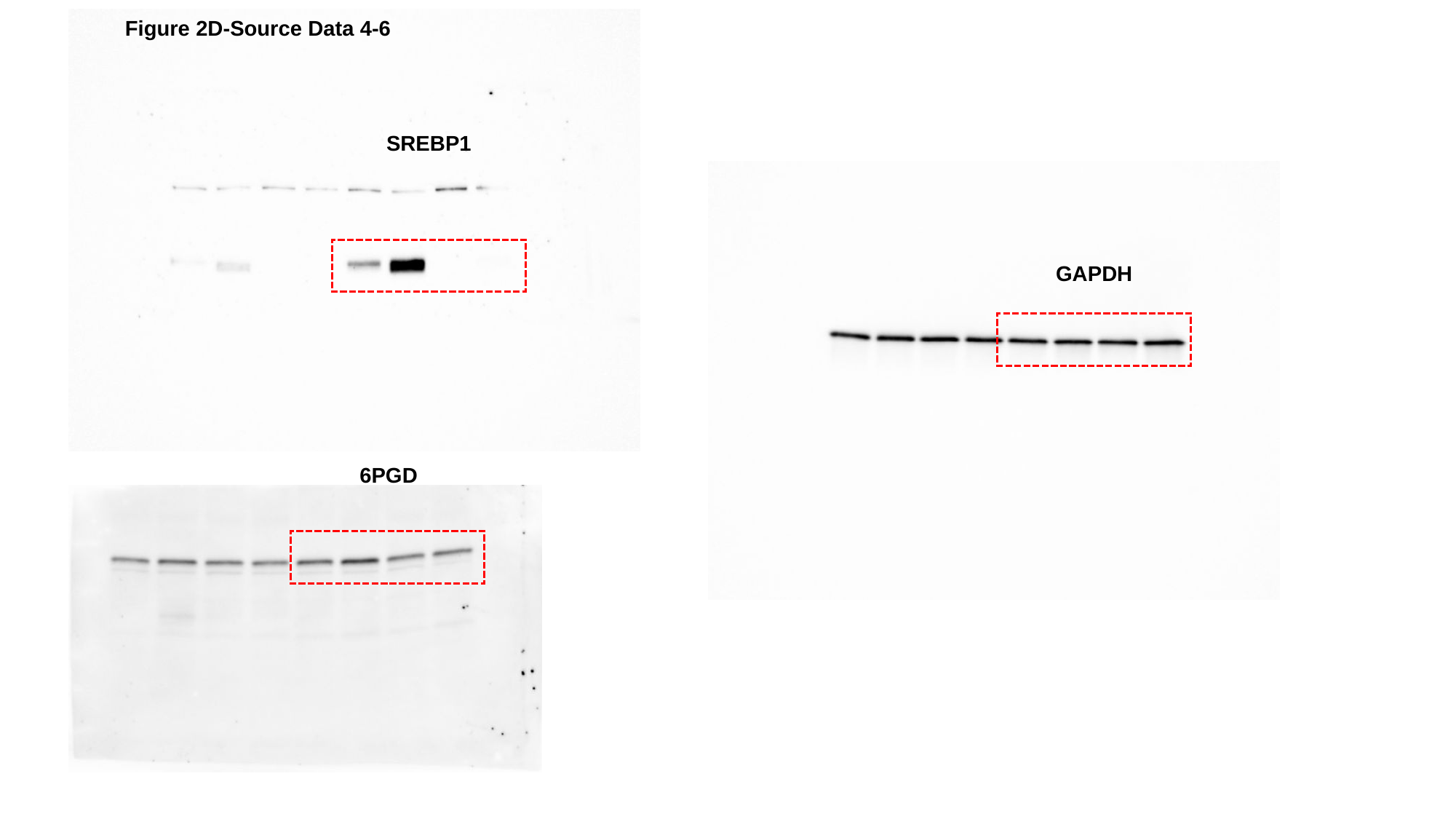

Figure 2D-Source Data 4-6
SREBP1
GAPDH
6PGD

## Slide 10
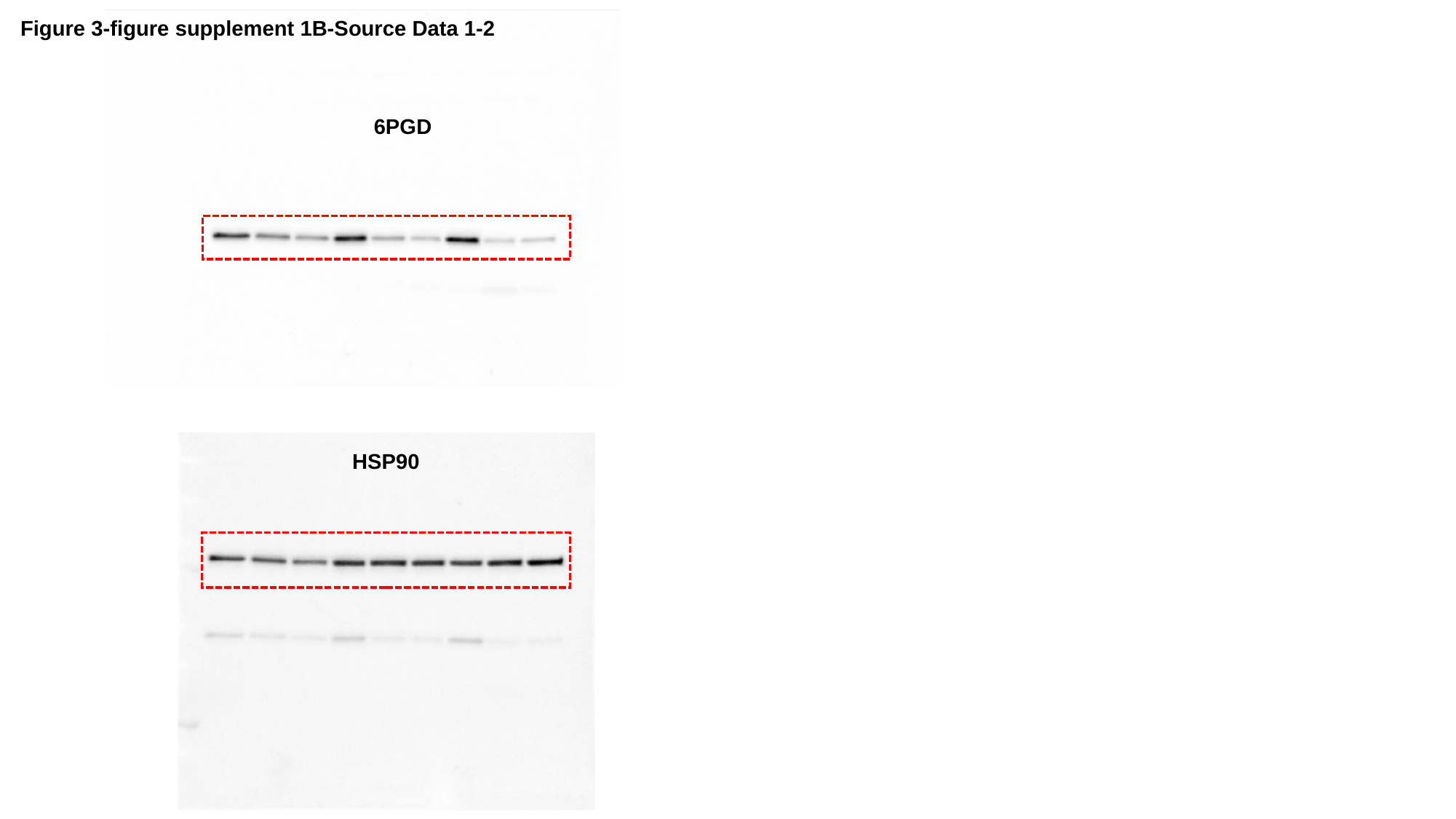

Figure 3-figure supplement 1B-Source Data 1-2
6PGD
HSP90

## Slide 11
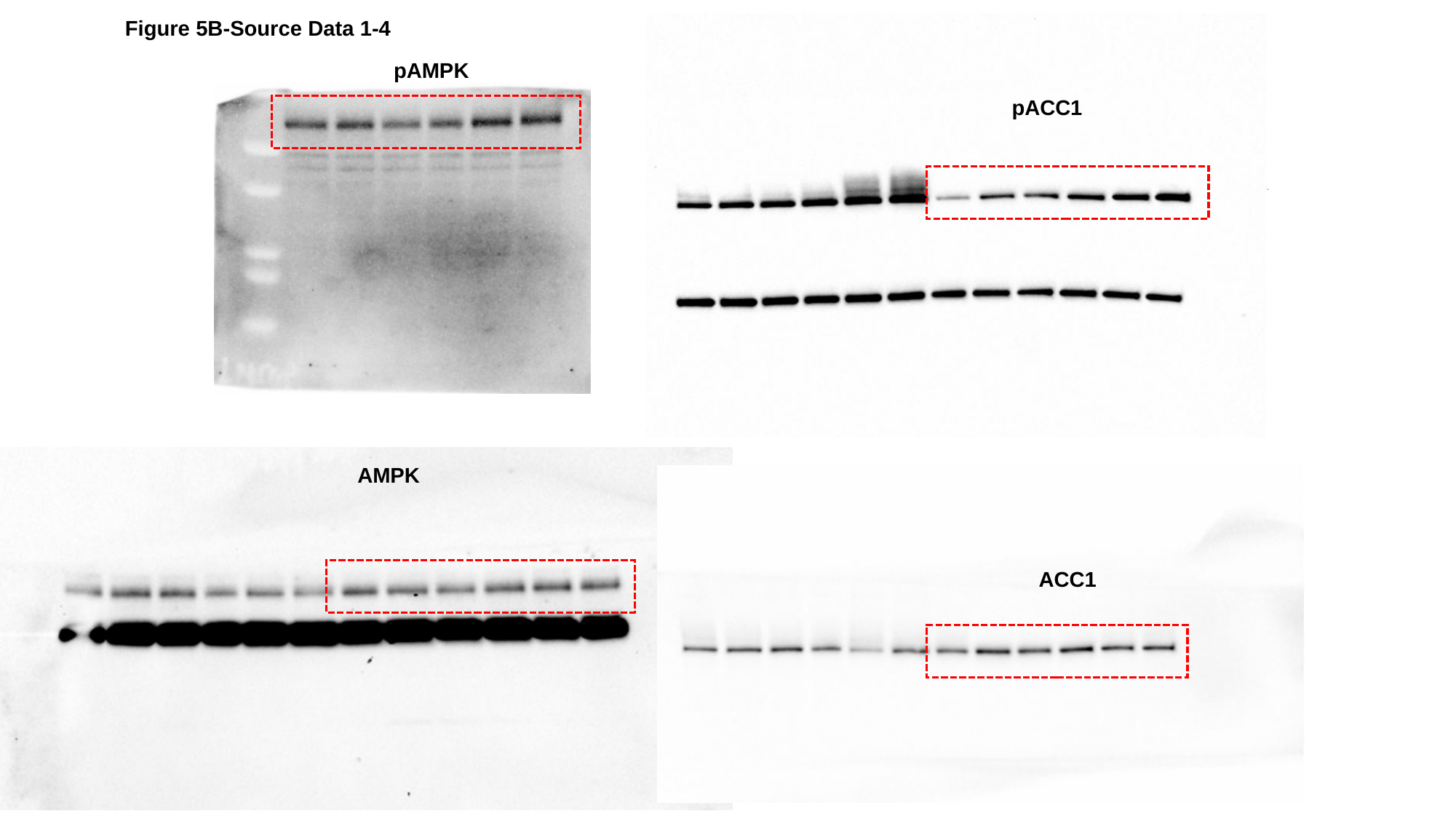

Figure 5B-Source Data 1-4
pAMPK
pACC1
AMPK
ACC1

## Slide 12
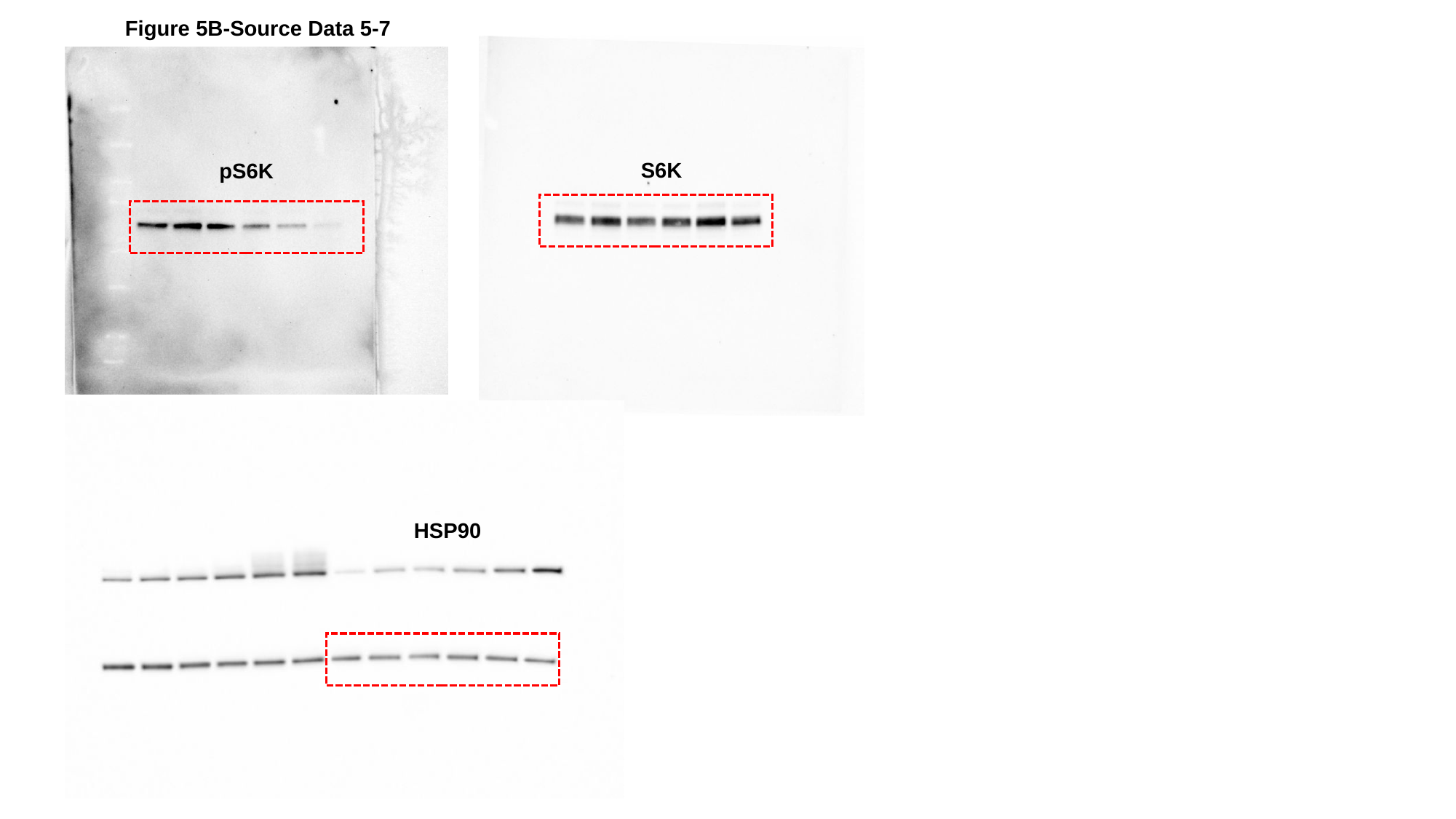

Figure 5B-Source Data 5-7
S6K
pS6K
HSP90

## Slide 13
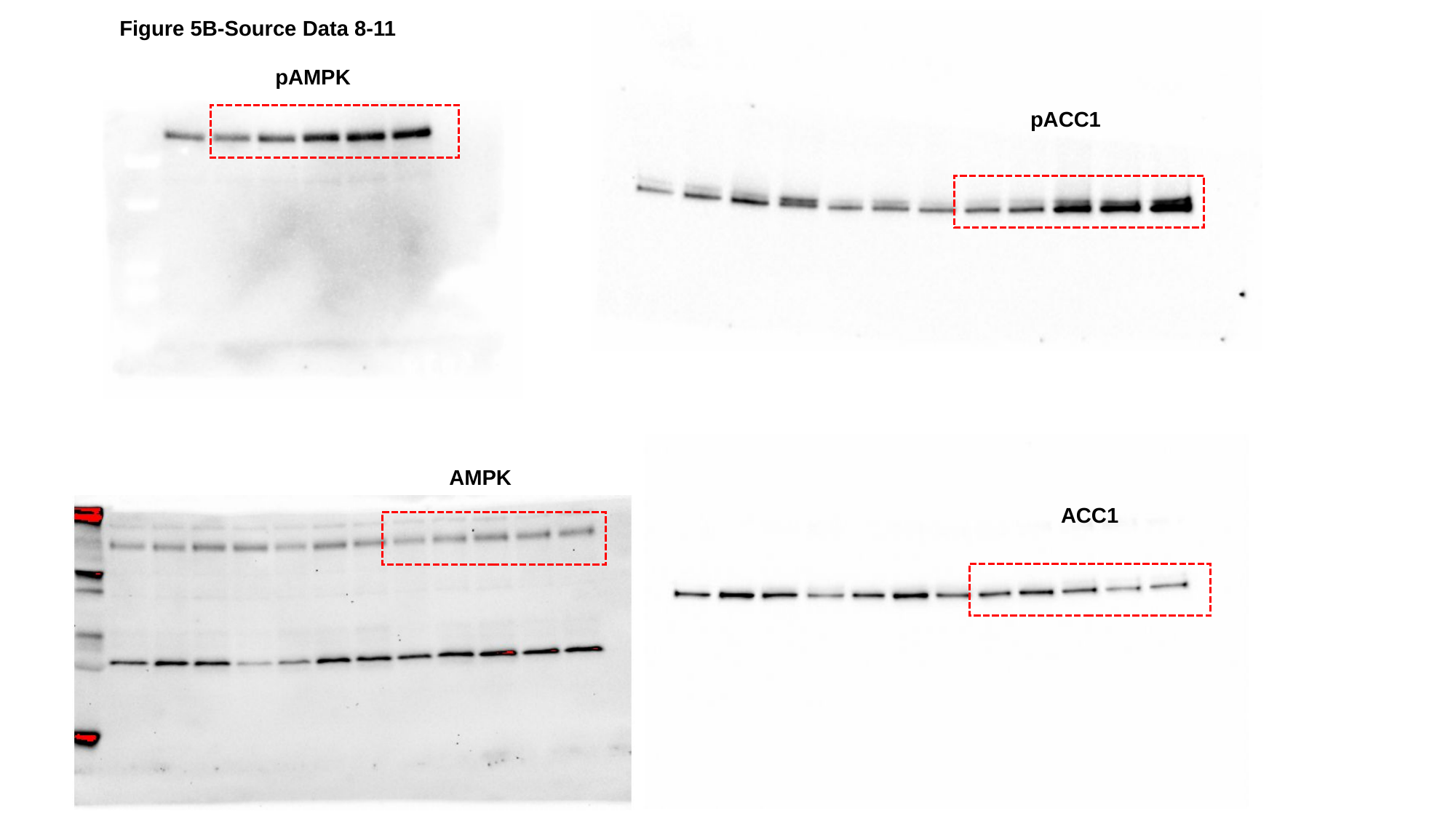

Figure 5B-Source Data 8-11
pAMPK
pACC1
AMPK
ACC1

## Slide 14
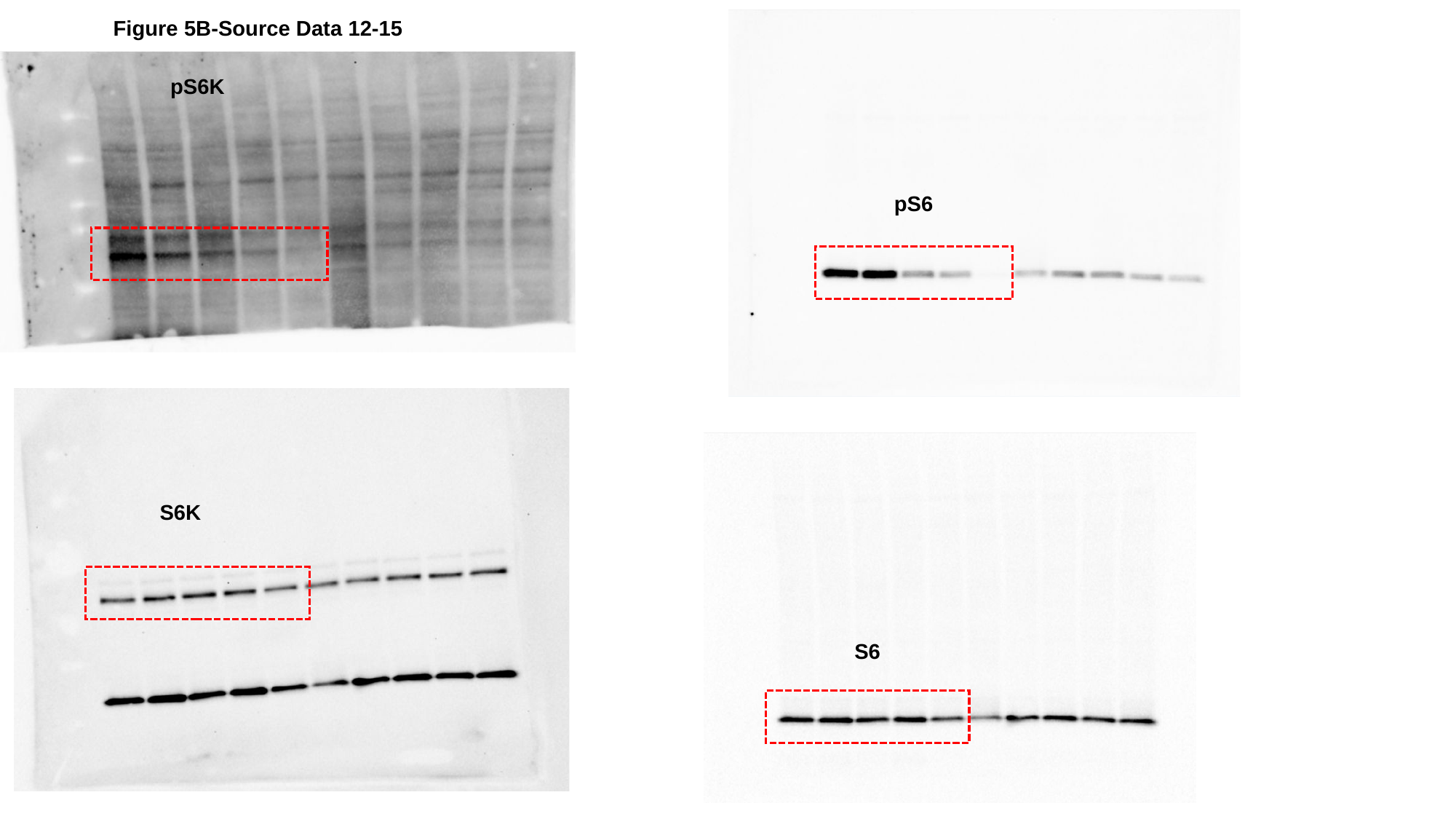

Figure 5B-Source Data 12-15
pS6K
pS6
S6K
S6

## Slide 15
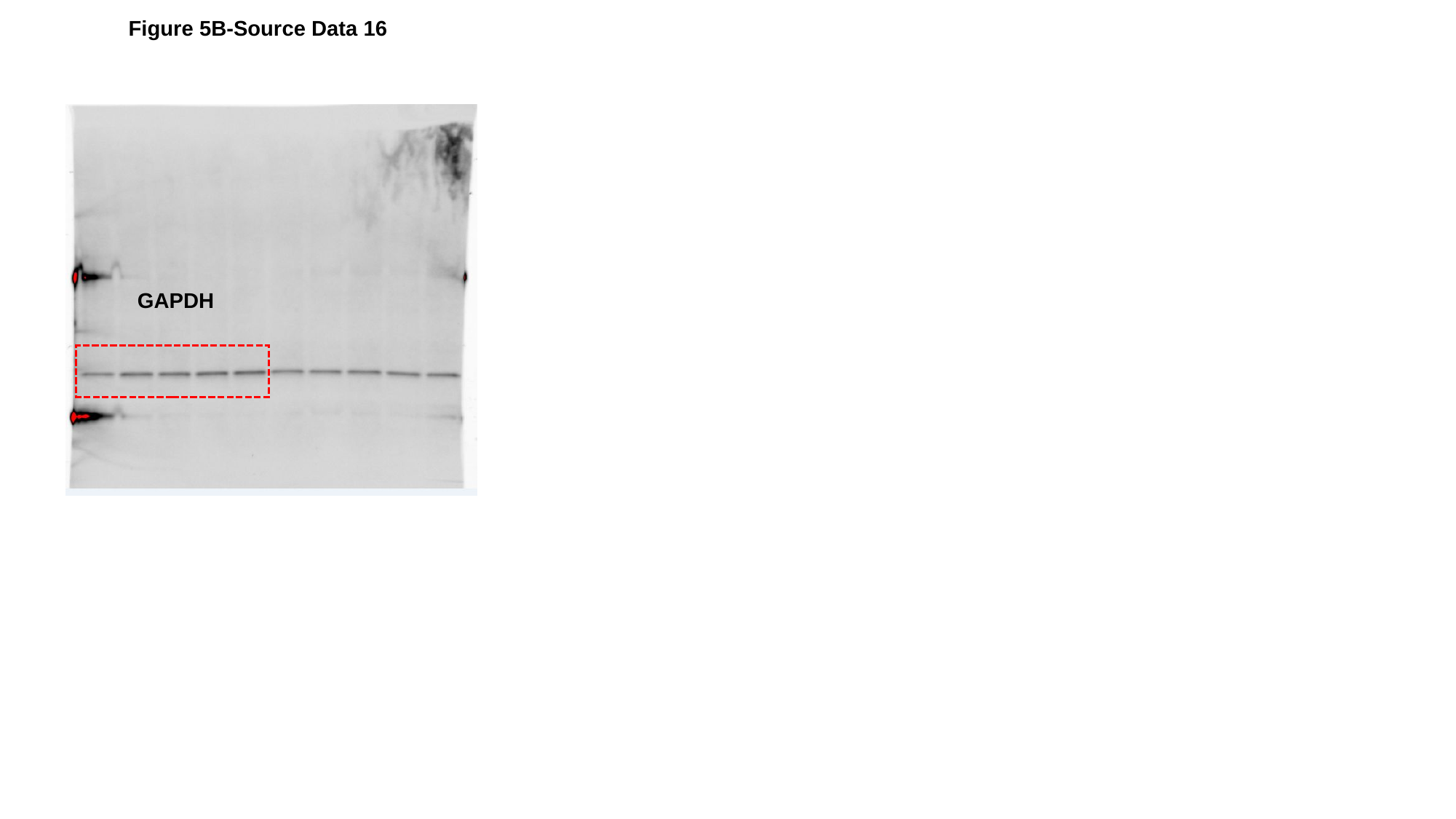

Figure 5B-Source Data 16
GAPDH

## Slide 16
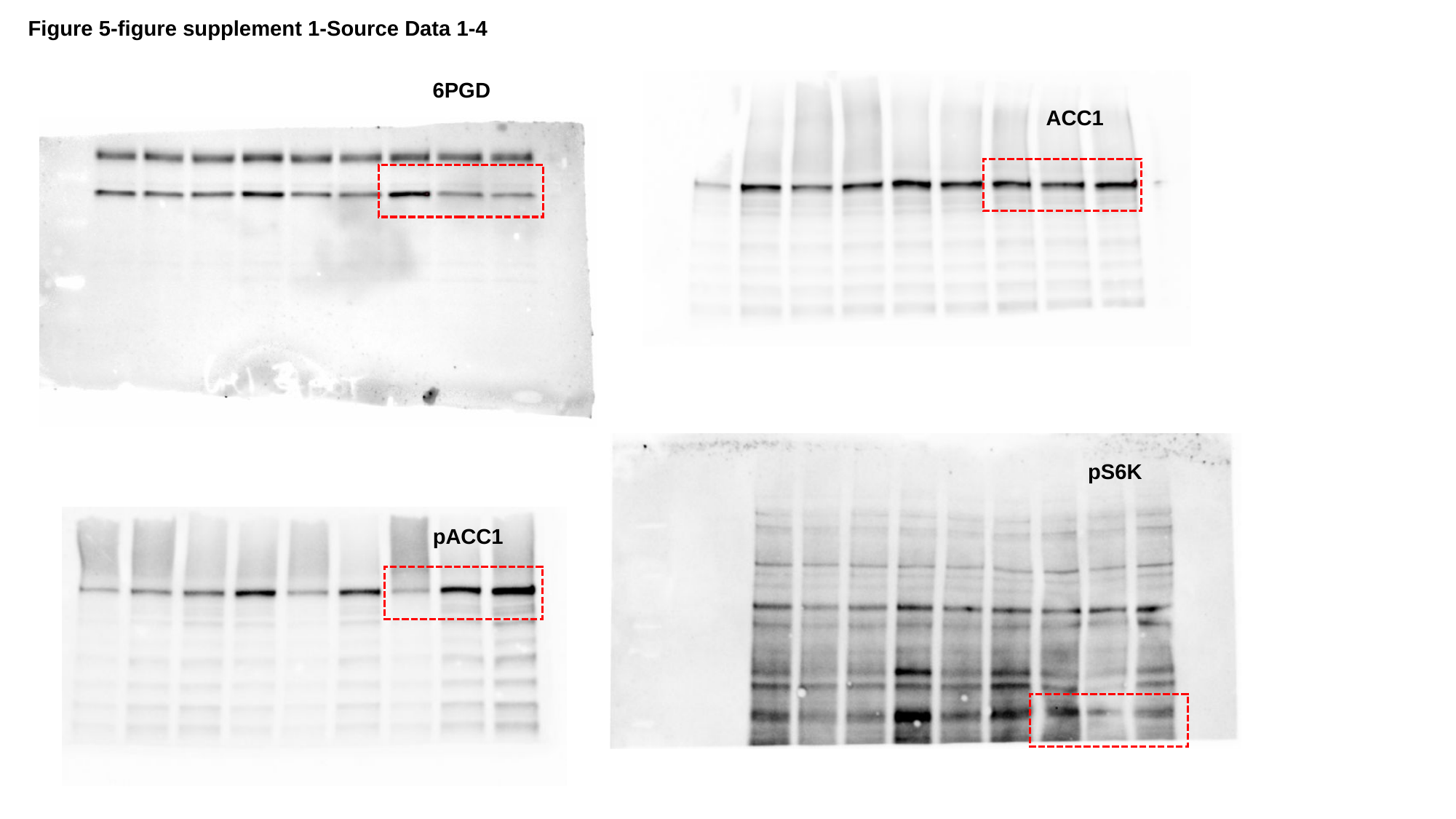

Figure 5-figure supplement 1-Source Data 1-4
6PGD
ACC1
pS6K
pACC1

## Slide 17
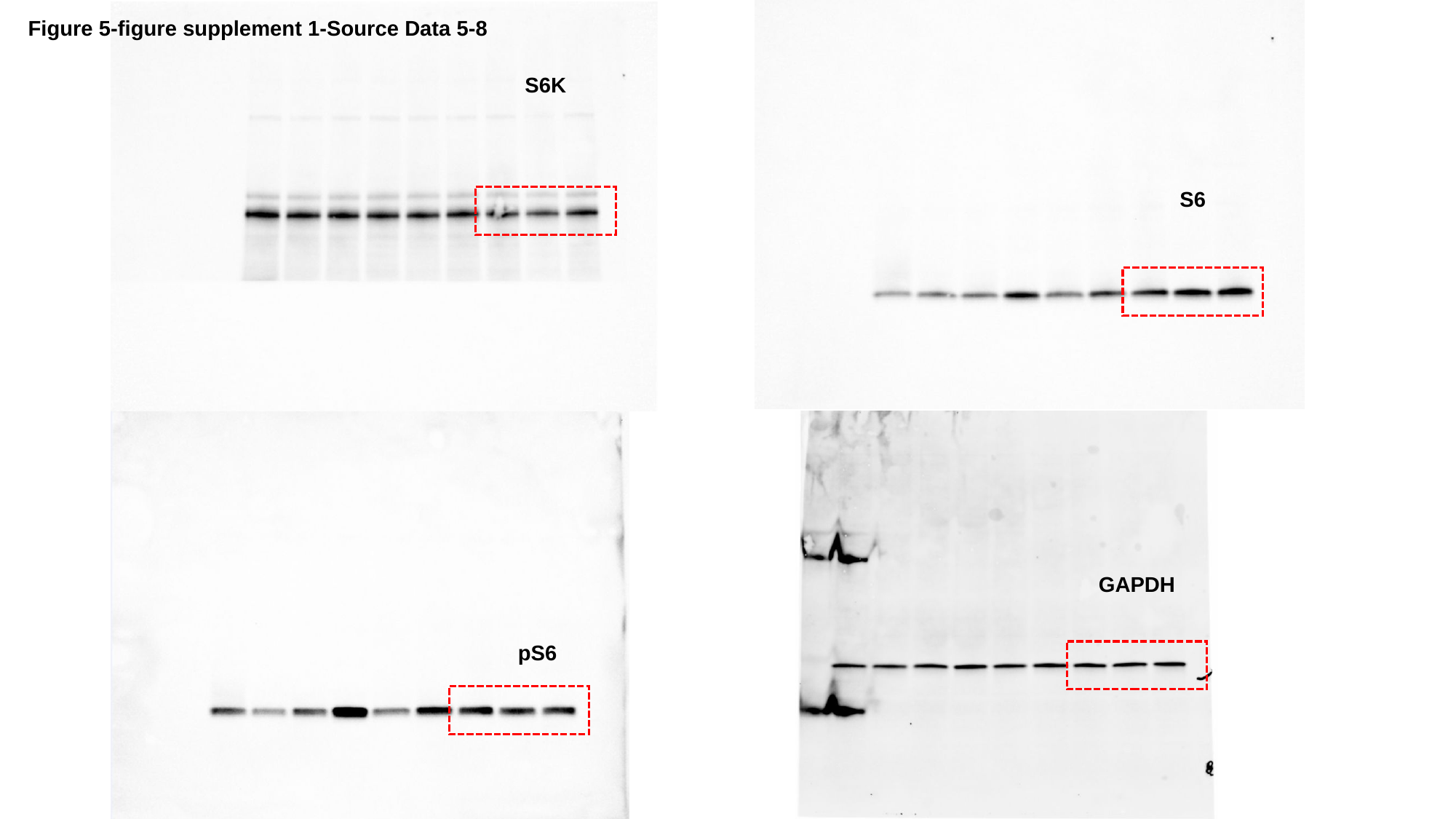

Figure 5-figure supplement 1-Source Data 5-8
S6K
S6
GAPDH
pS6

## Slide 18
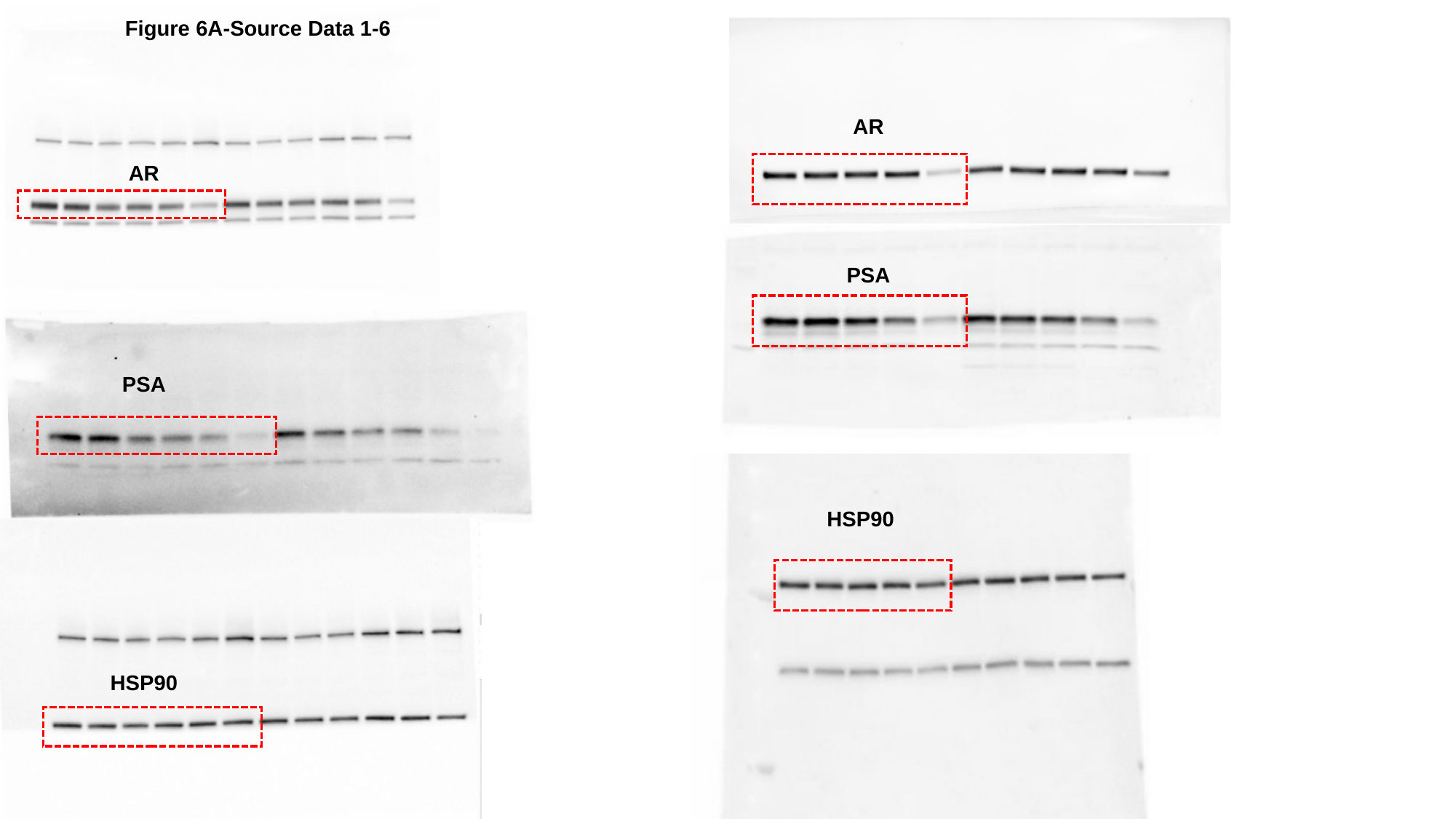

Figure 6A-Source Data 1-6
AR
AR
PSA
PSA
HSP90
HSP90

## Slide 19
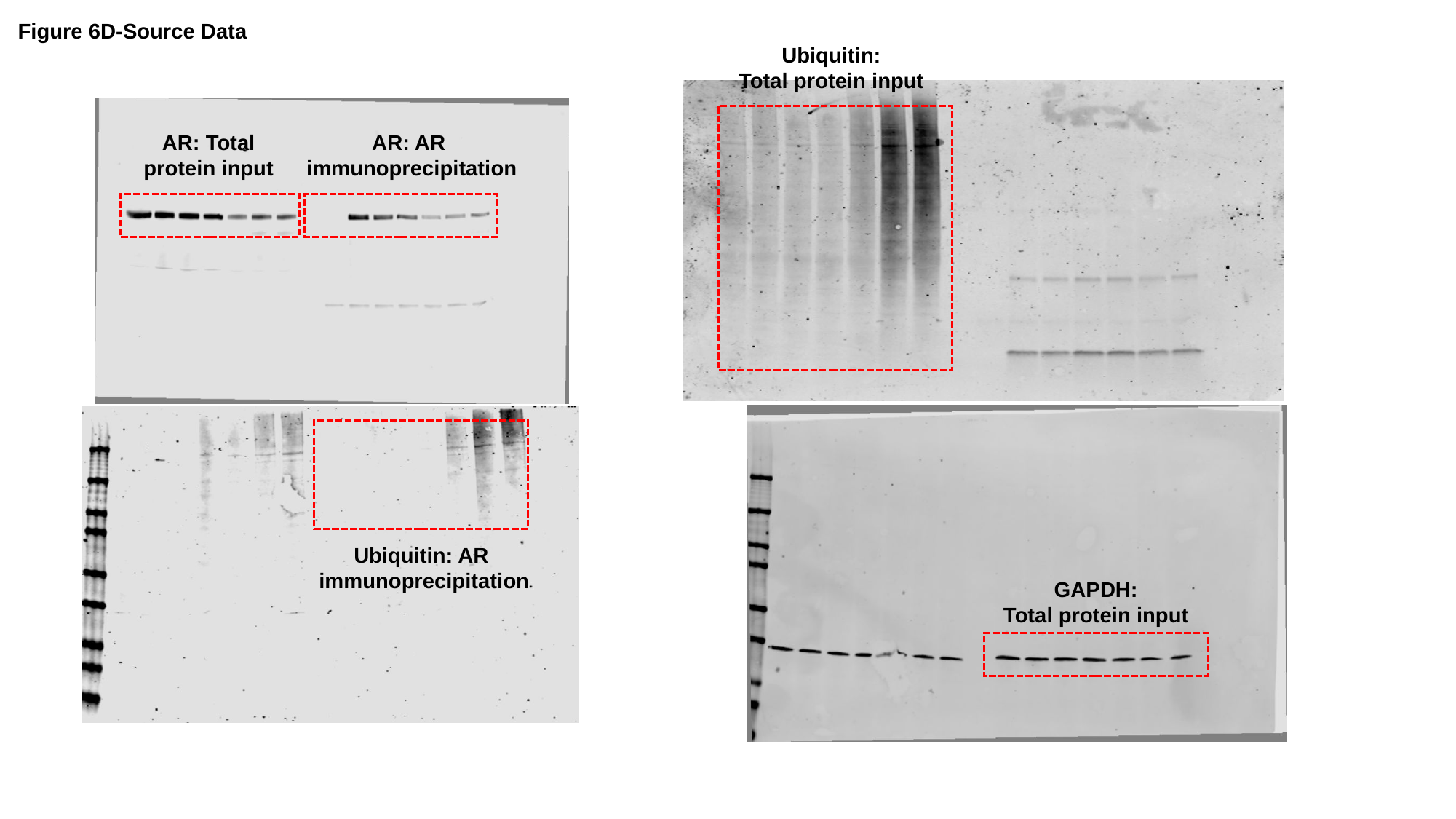

Figure 6D-Source Data
Ubiquitin:
Total protein input
AR: Total
protein input
AR: AR
immunoprecipitation
AR
Ubiquitin: AR
immunoprecipitation
GAPDH:
Total protein input

## Slide 20
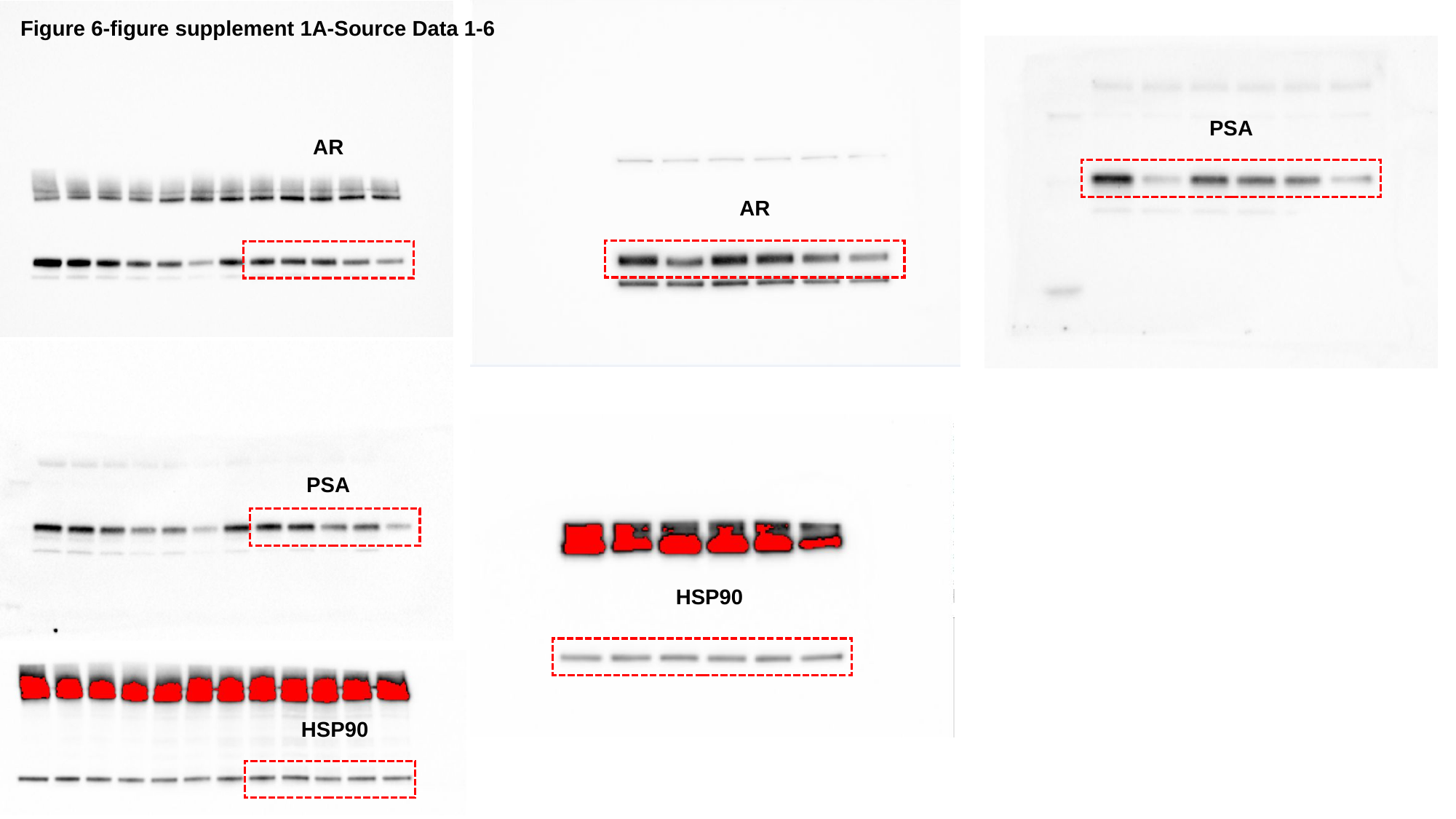

Figure 6-figure supplement 1A-Source Data 1-6
PSA
AR
AR
PSA
HSP90
HSP90
