# Supplementary figures and images for: A feedback loop between the androgen receptor and 6-phosphogluoconate dehydrogenase (6PGD) drives prostate cancer growth
Source: eLife. 2021 Aug 12;10:e62592. doi: 10.7554/eLife.62592 (PMC8416027; doi:10.7554/eLife.62592)

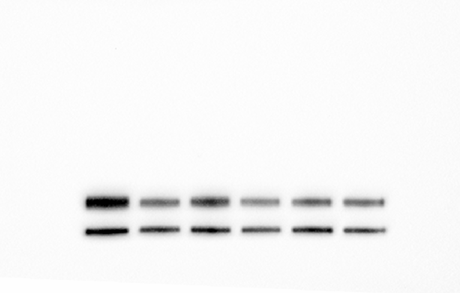

Supplement: Source data 1. [file elife-62592-supp3.zip › Figure 1-figure supplement 1D-Source Data 1 Apalutamide, AR).png]

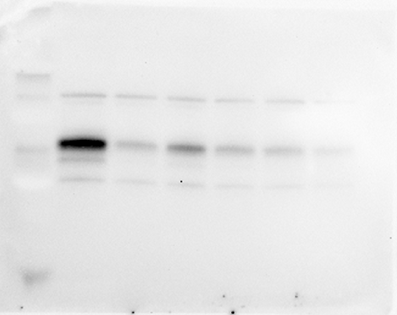

Supplement: Source data 1. [file elife-62592-supp3.zip › Figure 1-figure supplement 1D-Source Data 2 (Apalutamide, PSA).png]

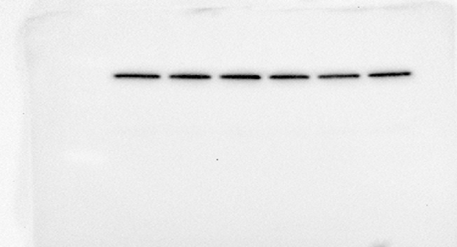

Supplement: Source data 1. [file elife-62592-supp3.zip › Figure 1-figure supplement 1D-Source Data 3 (Apalutamide, 6PGD).png]

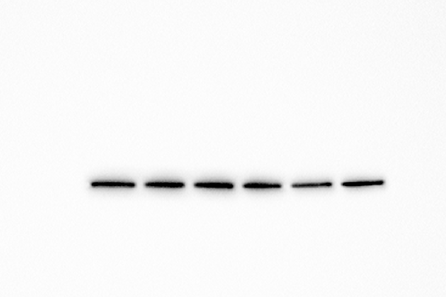

Supplement: Source data 1. [file elife-62592-supp3.zip › Figure 1-figure supplement 1D-Source Data 4 (Apalutamide, B-actin).png]

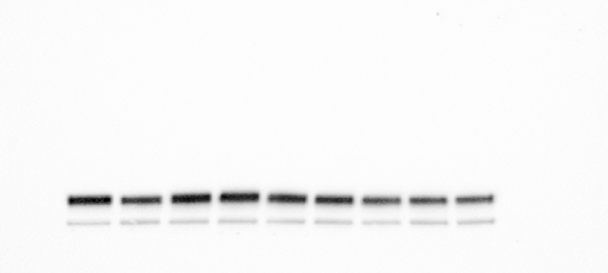

Supplement: Source data 1. [file elife-62592-supp3.zip › Figure 1-figure supplement 1D-Source Data 5 (Darolutamide, AR).png]

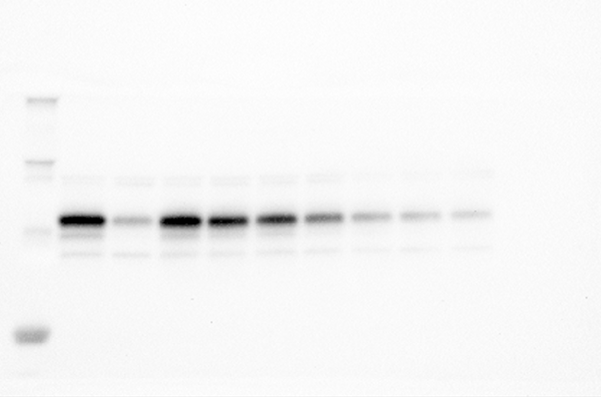

Supplement: Source data 1. [file elife-62592-supp3.zip › Figure 1-figure supplement 1D-Source Data 6 (Darolutamide, PSA).png]

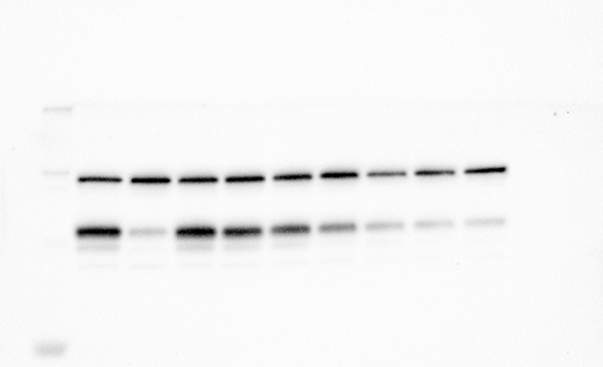

Supplement: Source data 1. [file elife-62592-supp3.zip › Figure 1-figure supplement 1D-Source Data 7 (Darolutamide, 6PGD).png]

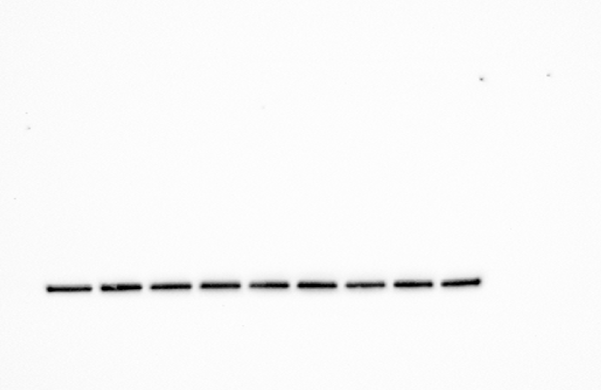

Supplement: Source data 1. [file elife-62592-supp3.zip › Figure 1-figure supplement 1D-Source Data 8 (Darolutamide, Hsp90).png]

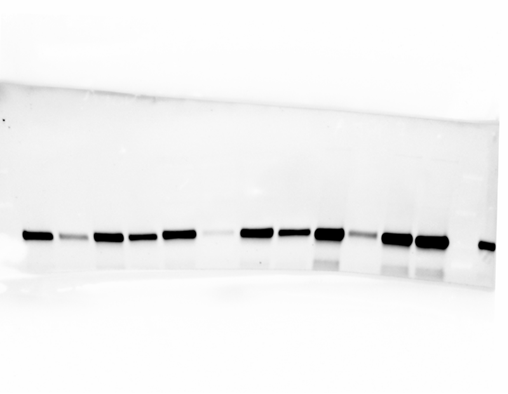

Supplement: Source data 1. [file elife-62592-supp3.zip › Figure 1A-Source data 1 (AR).png]

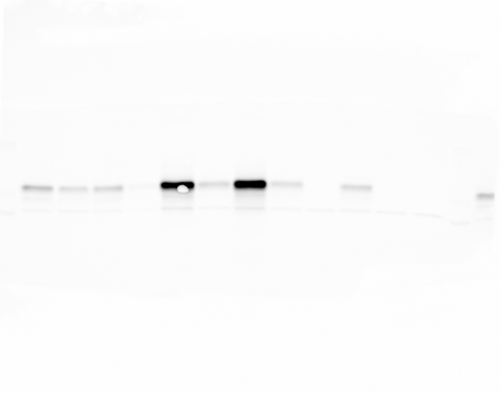

Supplement: Source data 1. [file elife-62592-supp3.zip › Figure 1A-Source data 2 (PSA).png]

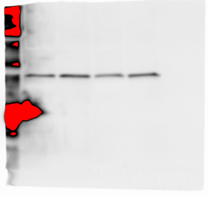

Supplement: Source data 1. [file elife-62592-supp3.zip › Figure 1A-Source data 3 (GAPDH).png]

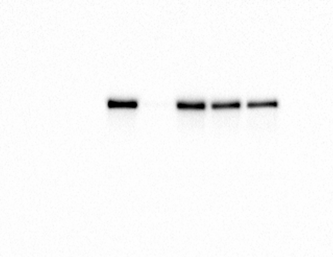

Supplement: Source data 1. [file elife-62592-supp3.zip › Figure 1E-Source data 1 (AR).png]

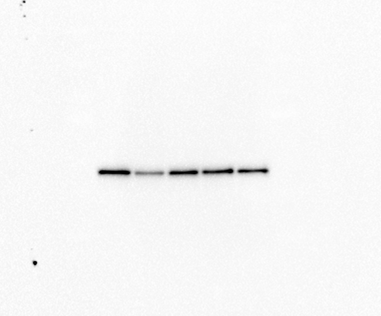

Supplement: Source data 1. [file elife-62592-supp3.zip › Figure 1E-Source data 2 (PGD).png]

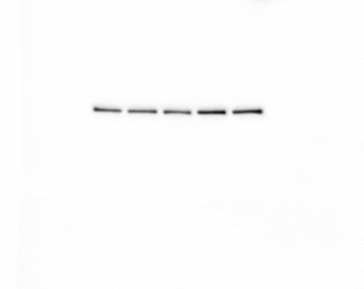

Supplement: Source data 1. [file elife-62592-supp3.zip › Figure 1E-Source data 3 (HSP90).png]

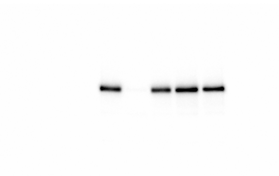

Supplement: Source data 1. [file elife-62592-supp3.zip › Figure 1E-Source data 4 (AR).png]

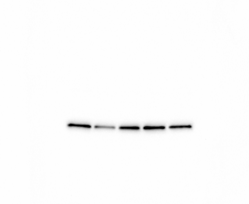

Supplement: Source data 1. [file elife-62592-supp3.zip › Figure 1E-Source data 5 (PGD).png]

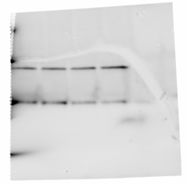

Supplement: Source data 1. [file elife-62592-supp3.zip › Figure 1E-Source data 6 (GAPDH).png]

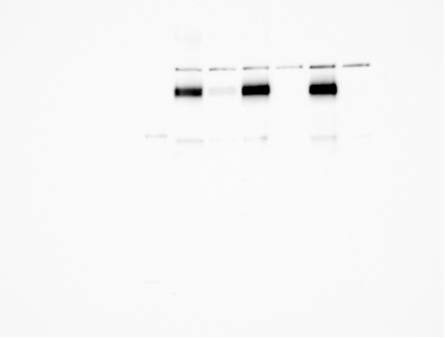

Supplement: Source data 1. [file elife-62592-supp3.zip › Figure 2C-Source data 1 (SREBP1).png]

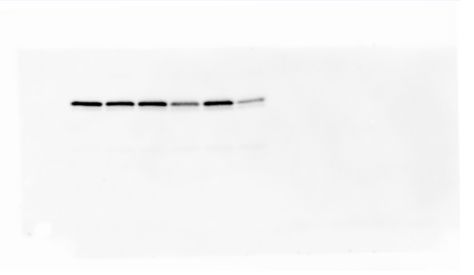

Supplement: Source data 1. [file elife-62592-supp3.zip › Figure 2C-Source data 2 (PGD).png]

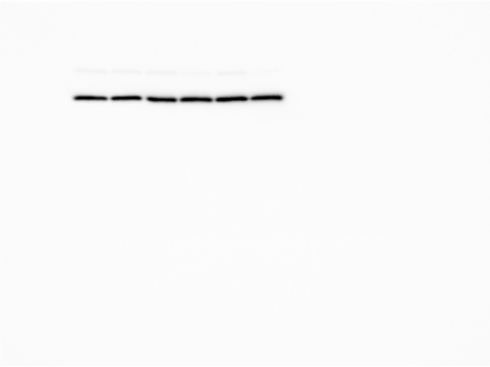

Supplement: Source data 1. [file elife-62592-supp3.zip › Figure 2C-Source data 3 (GAPDH).png]

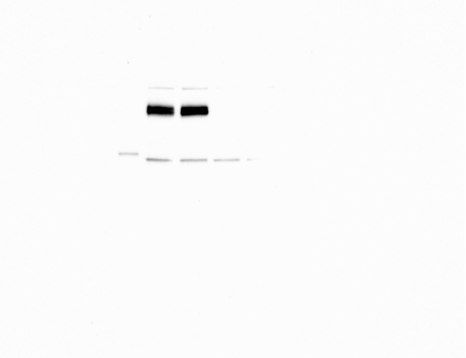

Supplement: Source data 1. [file elife-62592-supp3.zip › Figure 2D-Source data 1 (SREBP).png]

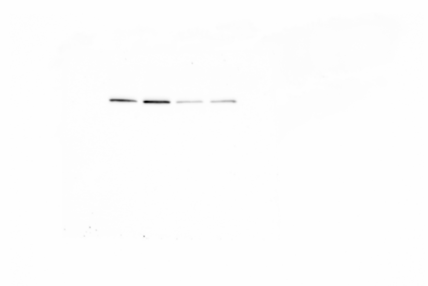

Supplement: Source data 1. [file elife-62592-supp3.zip › Figure 2D-Source data 2 (PGD).png]

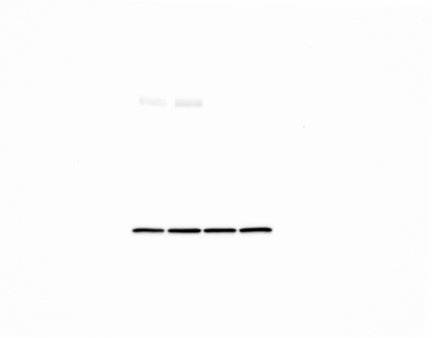

Supplement: Source data 1. [file elife-62592-supp3.zip › Figure 2D-Source data 3 (GAPDH).png]

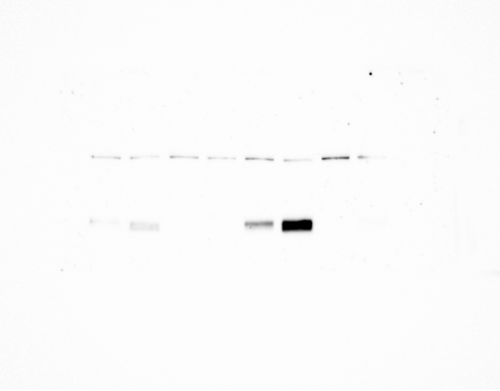

Supplement: Source data 1. [file elife-62592-supp3.zip › Figure 2D-Source data 4 (SREBP).png]

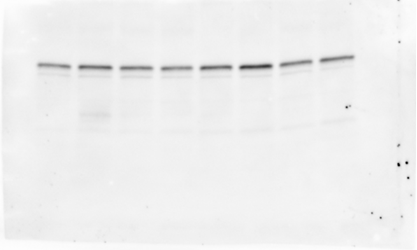

Supplement: Source data 1. [file elife-62592-supp3.zip › Figure 2D-Source data 5 (PGD).png]

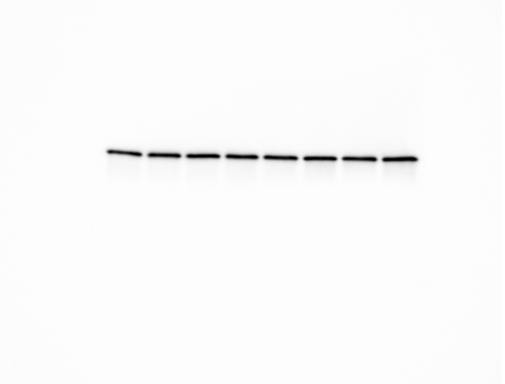

Supplement: Source data 1. [file elife-62592-supp3.zip › Figure 2D-Source data 6 (GAPDH).png]

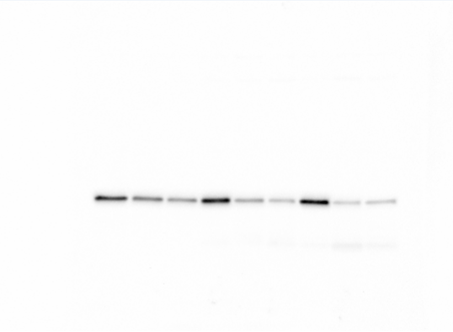

Supplement: Source data 1. [file elife-62592-supp3.zip › Figure 3-figure supplement 1B-Source data 1 (PGD).png]

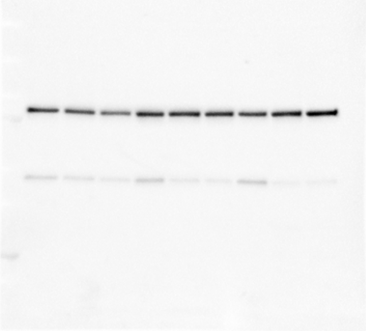

Supplement: Source data 1. [file elife-62592-supp3.zip › Figure 3-figure supplement 1B-Source data 2 (HSP90).png]

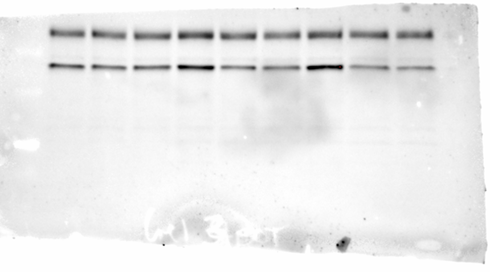

Supplement: Source data 1. [file elife-62592-supp3.zip › Figure 5-figure supplement 1-Source data 1 (PGD).png]

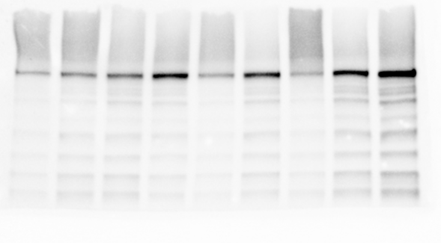

Supplement: Source data 1. [file elife-62592-supp3.zip › Figure 5-figure supplement 1-Source data 2 (pACC1).png]

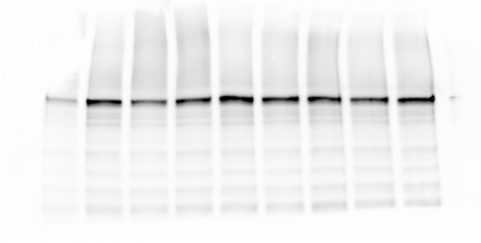

Supplement: Source data 1. [file elife-62592-supp3.zip › Figure 5-figure supplement 1-Source data 3 (ACC1).png]

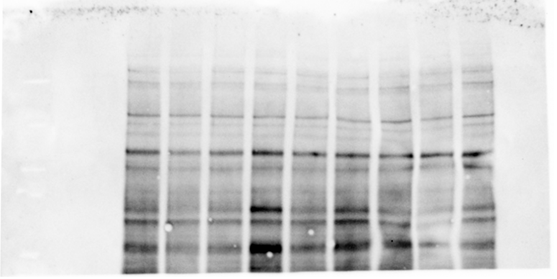

Supplement: Source data 1. [file elife-62592-supp3.zip › Figure 5-figure supplement 1-Source data 4 (pS6K).png]

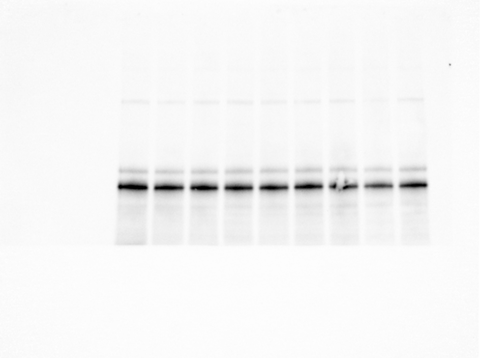

Supplement: Source data 1. [file elife-62592-supp3.zip › Figure 5-figure supplement 1-Source data 5 (S6K).png]

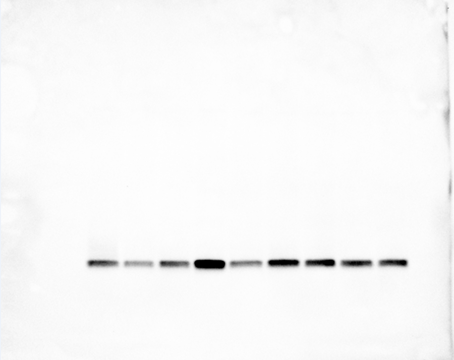

Supplement: Source data 1. [file elife-62592-supp3.zip › Figure 5-figure supplement 1-Source data 6 (pS6).png]

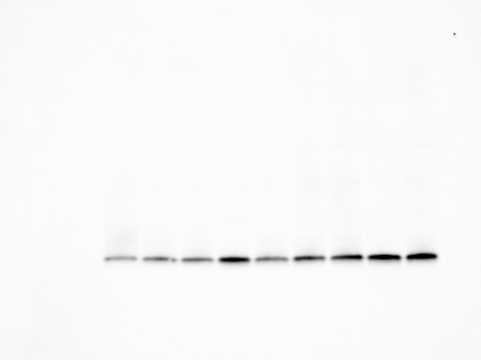

Supplement: Source data 1. [file elife-62592-supp3.zip › Figure 5-figure supplement 1-Source data 7 (S6).png]

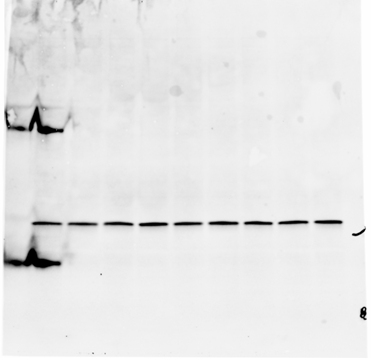

Supplement: Source data 1. [file elife-62592-supp3.zip › Figure 5-figure supplement 1-Source data 8 (GAPDH).png]

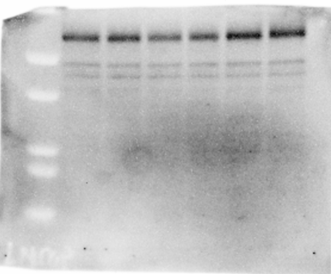

Supplement: Source data 1. [file elife-62592-supp3.zip › Figure 5B-Source data 1 (pAMPK).png]

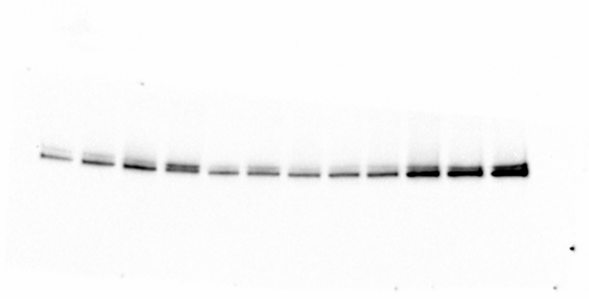

Supplement: Source data 1. [file elife-62592-supp3.zip › Figure 5B-Source data 10 (pACC1).png]

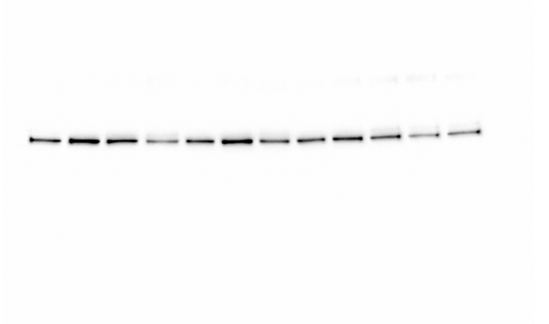

Supplement: Source data 1. [file elife-62592-supp3.zip › Figure 5B-Source data 11 (ACC1).png]

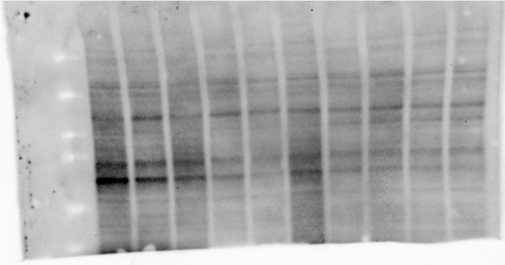

Supplement: Source data 1. [file elife-62592-supp3.zip › Figure 5B-Source data 12 (pS6K).png]

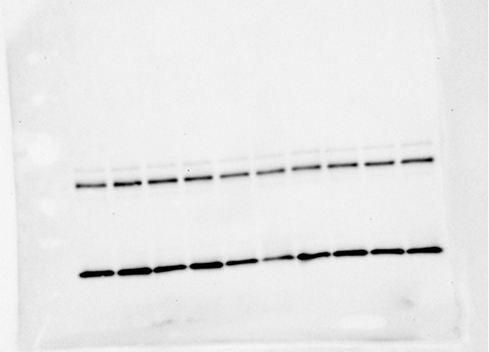

Supplement: Source data 1. [file elife-62592-supp3.zip › Figure 5B-Source data 13 (S6K).png]

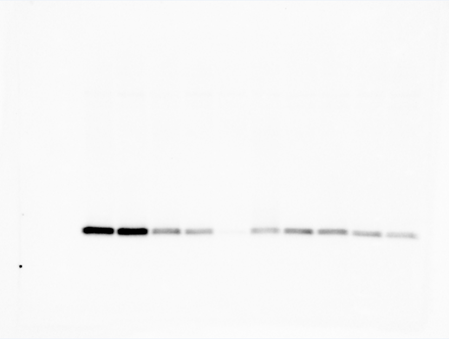

Supplement: Source data 1. [file elife-62592-supp3.zip › Figure 5B-Source data 14 (pS6).png]

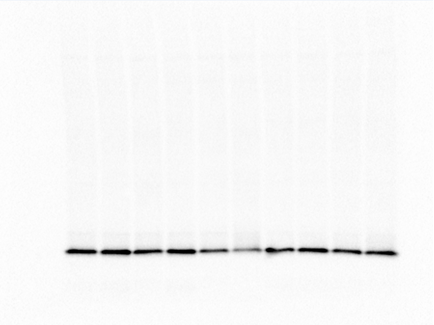

Supplement: Source data 1. [file elife-62592-supp3.zip › Figure 5B-Source data 15 (S6).png]

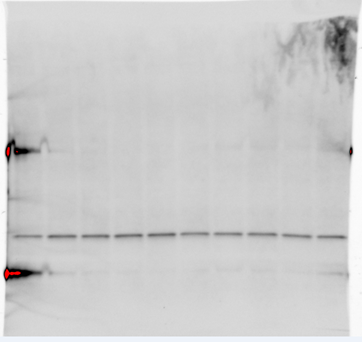

Supplement: Source data 1. [file elife-62592-supp3.zip › Figure 5B-Source data 16 (GAPDH).png]

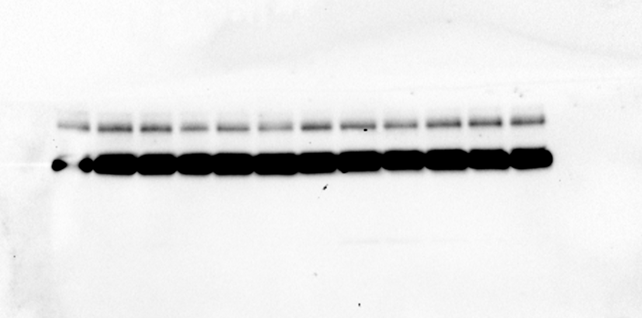

Supplement: Source data 1. [file elife-62592-supp3.zip › Figure 5B-Source data 2 (AMPK).png]

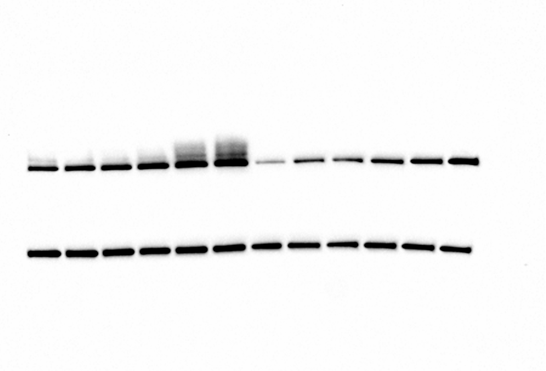

Supplement: Source data 1. [file elife-62592-supp3.zip › Figure 5B-Source data 3 (pACC1).png]

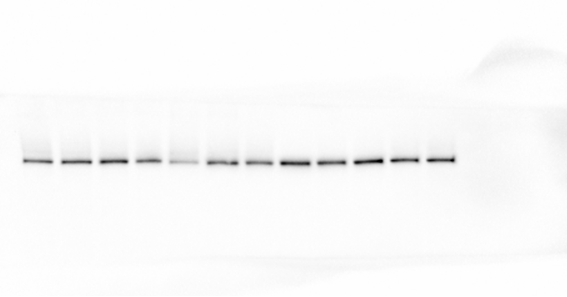

Supplement: Source data 1. [file elife-62592-supp3.zip › Figure 5B-Source data 4 (ACC1).png]

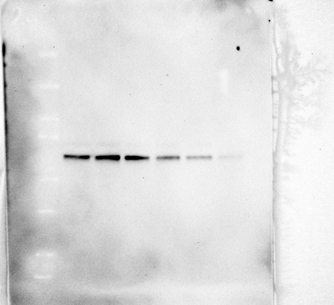

Supplement: Source data 1. [file elife-62592-supp3.zip › Figure 5B-Source data 5 (pS6K).png]

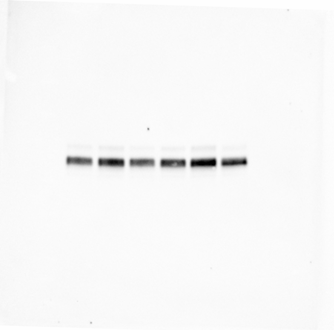

Supplement: Source data 1. [file elife-62592-supp3.zip › Figure 5B-Source data 6 (S6K).png]

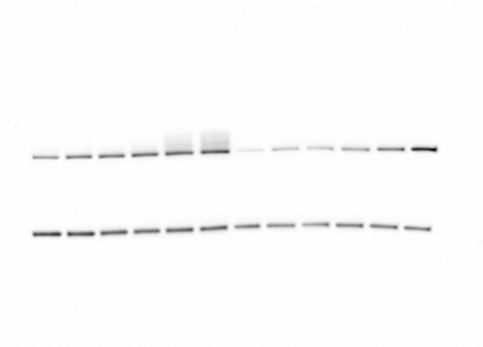

Supplement: Source data 1. [file elife-62592-supp3.zip › Figure 5B-Source data 7 (HSP90).png]

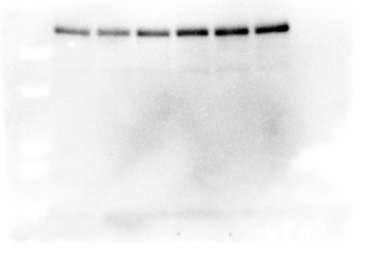

Supplement: Source data 1. [file elife-62592-supp3.zip › Figure 5B-Source data 8 (pAMPK).png]

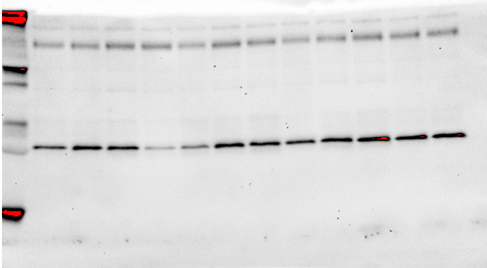

Supplement: Source data 1. [file elife-62592-supp3.zip › Figure 5B-Source data 9 (AMPK).png]

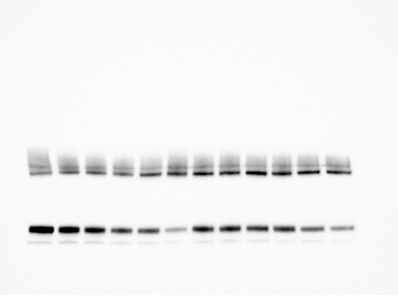

Supplement: Source data 1. [file elife-62592-supp3.zip › Figure 6-figure supplement 1-Source data 1 (AR).png]

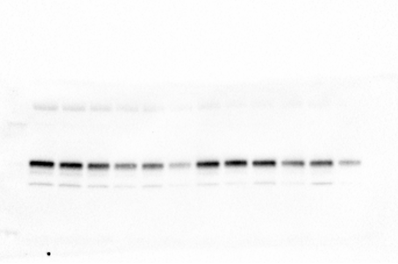

Supplement: Source data 1. [file elife-62592-supp3.zip › Figure 6-figure supplement 1-Source data 2 (PSA).png]

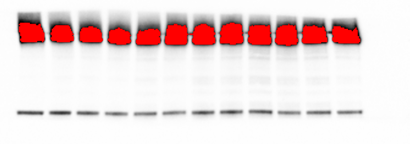

Supplement: Source data 1. [file elife-62592-supp3.zip › Figure 6-figure supplement 1-Source data 3 (HSP90).png]

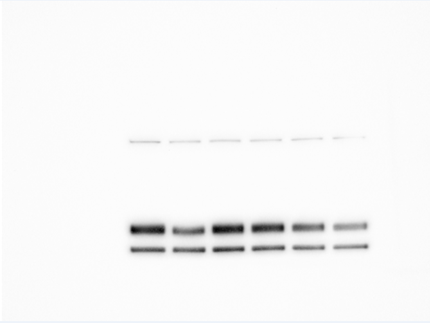

Supplement: Source data 1. [file elife-62592-supp3.zip › Figure 6-figure supplement 1-Source data 4 (AR).png]

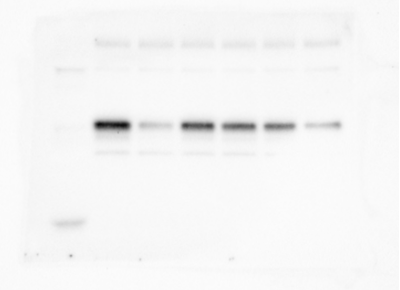

Supplement: Source data 1. [file elife-62592-supp3.zip › Figure 6-figure supplement 1-Source data 5 (PSA).png]

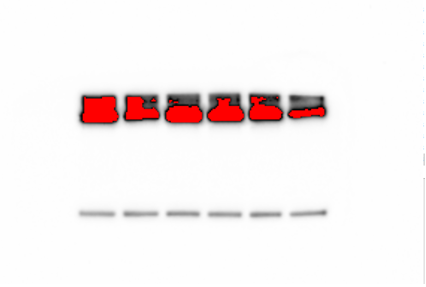

Supplement: Source data 1. [file elife-62592-supp3.zip › Figure 6-figure supplement 1-source data 6 (HSP90).png]

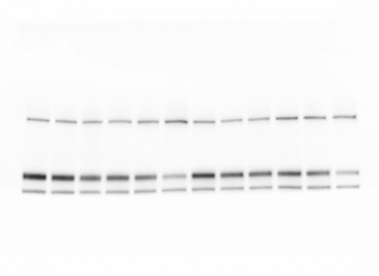

Supplement: Source data 1. [file elife-62592-supp3.zip › Figure 6A-Source data 1 (AR).png]

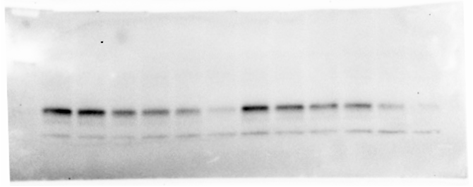

Supplement: Source data 1. [file elife-62592-supp3.zip › Figure 6A-Source data 2 (PSA).png]

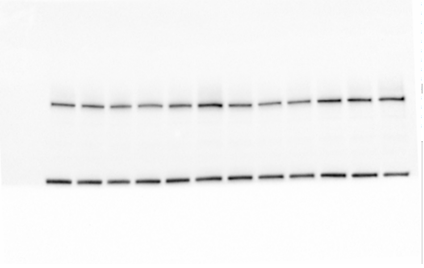

Supplement: Source data 1. [file elife-62592-supp3.zip › Figure 6A-Source data 3 (HSP90).png]

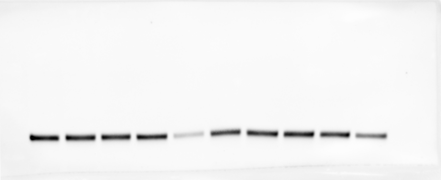

Supplement: Source data 1. [file elife-62592-supp3.zip › Figure 6A-Source data 4 (AR).png]

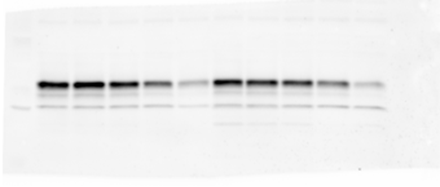

Supplement: Source data 1. [file elife-62592-supp3.zip › Figure 6A-Source data 5 (PSA).png]

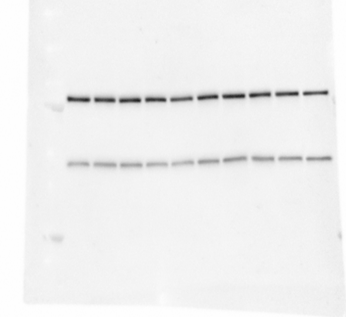

Supplement: Source data 1. [file elife-62592-supp3.zip › Figure 6A-Source data 6 (HSP90).png]

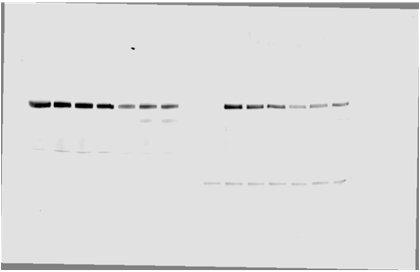

Supplement: Source data 1. [file elife-62592-supp3.zip › Figure 6D_Source data 1,3 (AR).png]

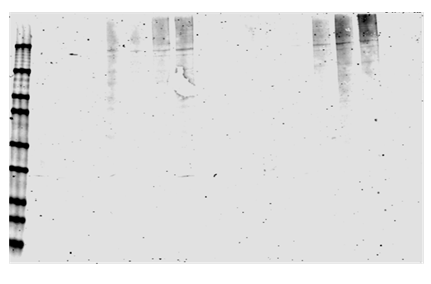

Supplement: Source data 1. [file elife-62592-supp3.zip › Figure 6D_Source data 2 (Ubiquitin).png]

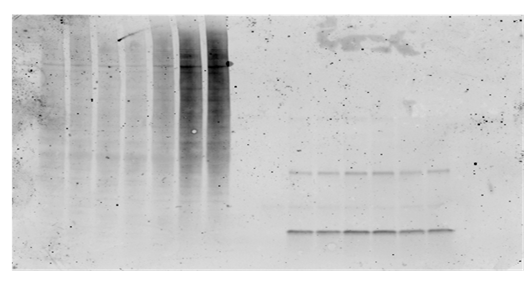

Supplement: Source data 1. [file elife-62592-supp3.zip › Figure 6D_Source data 4 (Ubiquitin).png]

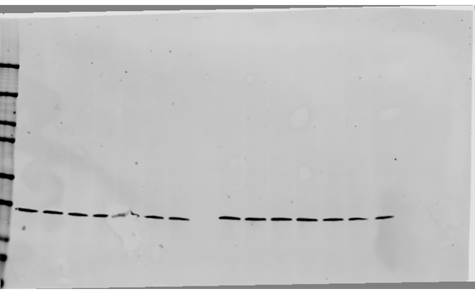

Supplement: Source data 1. [file elife-62592-supp3.zip › Figure 6D_Source data 5 (GAPDH).png]
